# Burden and trends of stroke attributable to dietary risk factors from 1990 to 2019 in the Belt and Road Initiative countries: an analysis from the global burden of disease study 2019

**DOI:** 10.3389/fnut.2023.1235271

**Published:** 2023-07-26

**Authors:** Yue Zhang, Zheng Luo, Juan Yi, Junjie Zhu, Yun Qiu, Xiaoyun Xu, Wanying Xie, Jinyi Wu, Huihui Lv, Changhua Mou, Wei Zhang, Xiaopan Li

**Affiliations:** ^1^Key Laboratory of Coal Environmental Pathogenicity and Prevention, Ministry Education, Department of Epidemiology, School of Public Health, Shanxi Medical University, Taiyuan, China; ^2^Department of Neurology, Shanghai University of Medicine and Health Sciences Affiliated Zhoupu Hospital, Shanghai, China; ^3^Department of Neurology, Zhuzhou Central Hospital, Zhuzhou, Hunan, China; ^4^Department of Epidemiology and Health Statistics, School of Public Health, Dali University, Dali, China; ^5^Department of Public Health, Nanjing University of Chinese Medicine, Nanjing, Jiangsu, China; ^6^Department of Traditional Chinese Medicine Encephalopathy, Shanghai Pudong Traditional Chinese Medicine Hospital, Shanghai, China; ^7^Department of Public Health, Wuhan Fourth Hospital, Wuhan, China; ^8^Department of Neurology, Yueyang Hospital of Integrated Traditional Chinese and Western Medicine, Shanghai University of Traditional Chinese Medicine, Shanghai, China; ^9^Department of Neurology, Shanghai Children’s Medical Center, Shanghai Jiao Tong University School of Medicine, Shanghai, China; ^10^Department of Health Management Center, Zhongshan Hospital, Shanghai Medical College of Fudan University, Shanghai, China

**Keywords:** “B&R” countries, stroke, burden of disease, dietary risk factors, disability-adjusted life years, average annual percent change, trend analysis

## Abstract

**Objectives:**

This study aimed to compare the burden and trends of stroke attributed to dietary risk factors in the Belt and Road (“B&R”) countries from 1990 to 2019.

**Methods:**

The 2019 Global Burden of Disease (GBD) Study was used to gather information on the burden of stroke attributable to dietary risk factors. Numbers and age-standardized rates (ASRs) of deaths, disability-adjusted life years (DALYs) were determined in 1990 and 2019 among the “B&R” countries. The average annual percent change (AAPC) was used to analyze the temporal trends of diet-induced stroke DALYs from 1990 to 2019 and in the final decade (2010–2019) by Joinpoint regression analysis.

**Results:**

In 2019, the absolute number of stroke deaths and DALYs attributable to dietary risk factors were 671,872 cases (95% UI 436,354–937,093) and 1.67 million cases (95% UI 1.15–2.24) in China. We found geographical differences in mortality and DALYs of diet-attributable stroke among member countries, with Bulgaria, Hungary and Serbia being the three highest countries in 1990, Bulgaria, North Macedonia and Montenegro in Central Asia in 2019. The ASRs of diet-induced stroke mortality and DALYs were generally declining in most member states from 1990 to 2019, however, the corresponding metrics in Mongolia remained high. The fastest decline in ASR of mortality and DALYs for diet-induced stroke was seen in Estonia, Eastern Europe, with AAPC values of −7.09% (95%CI: −7.72, −6.46%) and − 6.62% (95%CI: −7.20, −6.03%), respectively. We noted a substantial downward trend in ASR of mortality and DALYs from diet-induced stroke changes in the final decade (2010–2019) for most member states. The ASR of DALYs for diet-induced stroke decreased greater in females than in males. For those aged 50–74, the DALYs for stroke due to dietary risk factors in all other member countries of the “B&R” showed a decreasing trend, except for the Philippines, which rose (AAPC = 2.13, 95%CI: 1.40–2.87%) and Turkmenistan, which remained stable (AAPC = 0.05, 95%CI: −0.43–0.33%).

**Conclusion:**

The burden of diet-induced stroke varies substantially across “B&R” countries and threaten public health, relevant evidence-based policies and interventions should be adopted to address the future burden of stroke in “B&R” countries through extensive collaboration.

## Introduction

Stroke is a global public health problem that imposes a heavy disease and financial burden on individuals and society. According to the 2019 Global Burden of Disease, Injury and Risk Factor Study (GBD 2019), stroke was the second-leading cause of death (11.6% of total deaths) and third-leading cause of disability (5.7% of total disability-adjusted life years [DALYs]) worldwide (1). Stroke incidence has maintained stable and mortality have declined over the last 20 year-period, however, DALYs due to stroke and stroke-related survivors have all increased, making stroke prevention a global health priority (2). American Heart Association (AHA) predicts that stroke incidence could reach 4% by 2030 among American adults, resulting in an increase in stroke-related medical costs to $183 billion (3). Additionally, GBD shows that more than 75% of stroke deaths and 80% of DALYs occur in low- and middle-income countries (1).

As we all know, health issues are no longer the responsibility of individual countries with the rapid pace of globalization. In 2013, Chinese government initiates the Belt and Road Initiative (BRI) to accelerate infrastructure, trade development and business partnerships among 66 countries in Asia, Europe, South America and Africa (4, 5). Although the BRI focuses on economic development and infrastructure investment, its impact on global health is emerging (6). In 2017, Chinese government launched the “Health Silk Road” (HSR) initiative to strengthen global health cooperation. Under the framework of HSR, a variety of regional and trans-regional plans have been implemented, including the training of health professionals, the establishment of disease control centers, and the creation of knowledge sharing networks. Through the HSR, China could use BRI transportation networks to provide health care and medical assistance to member countries. Against the backdrop of COVID-19, the BRI provides an important platform for member counties to discuss clinical treatment guidelines and epidemic control strategies (7, 8). Currently, member countries face the threat of stroke to varying degrees, and the distribution of risk factors is constantly changing, so analyzing the differences between member states is essential to allocate resources for prevention strategies (1, 9).

Recent epidemiological evidence has found that the large burden of stroke can be attributed to several modifiable factors, such as obesity, hypertension, diabetes, a sedentary lifestyle, or unhealthy diet (10). Diet is an important risk factor for stroke, a growing number of prospective observational studies have been performed to explore the impact of dietary factors on stroke risk (11). English et al. (12) found that poor diet and nutritional intake were strongly associated with the risk of first stroke, and a Mediterranean-style diet was reported to reduce the risk of first stroke. Baden et al. (13) explored the relationship between plant-based diet quality and total stroke risk and found that people who adhered to a healthy plant-based diet had a lower risk of total stroke.

The GBD 2019 framework, through extensive collection of data sources and statistical modeling, allows for comparable assessment of stroke burden in terms of mortality and DALYs. At present, none of the existing studies on stroke mortality and DALYs attributable to modifiable dietary risk factors had explored differences and the changing trend of DALYs stratified by gender, age and diet-specific risk factors among 66 countries from the BRI. Therefore, this study was conducted to compare the burden and trends of diet-induced stroke from 1990 to 2019 in the “B&R” countries, and to provide the basis for generating prevention and control strategies of stroke for building a healthy “B&R.”

## Methods

### Data sources

In this study, data on annual diet-induced stroke deaths, DALYs, and respective age-standardized rates (ASR) by gender, age and specific diet risk factor in the “B&R” countries from 1990 to 2019 were extracted from the GBD 2019 database.^1^ The GBD 2019, which is an international collaborative surveillance system, estimated 369 diseases and injuries, 87 risk factors and combinations of risk factors across 204 countries and territories from 1990 to 2019. It contains a total of 86,249 data input sources from censuses, household surveys, civil registration, vital statistics and other sources. The GBD 2019 Stroke Collaborators has presented methods for processing, standardizing, and modeling stroke mortality and DALYs (1). GBD estimates the burden of disease indices including incidence, prevalence, mortality, years lived with disability (YLD), years of life lost (YLL) and DALYs at regional, national and global levels. Detailed methodology has been published elsewhere (14, 15). The detailed information on the statistical codes for diet-related burden in the GBD study has been previously announced on the following website: http://ghdx.healthdata.org/gbd-2019/code/nonfatal-12. DALYs is a composite indicator to assess the disease burden of disability and premature death, which is obtained by summing YLL and YLD.

The composition of “B&R” countries is mainly based on the GBD classification of global regions and international political and economic organizations (16). BRI include 66 member countries, divided as follows: (1) East Asia: China, (2) Central Asia: Armenia, Azerbaijan, Georgia, Kazakhstan, Turkmenistan, Uzbekistan, Kyrgyzstan, Mongolia, Tajikistan, (3) South Asia: India, Nepal, Bangladesh, Bhutan, Pakistan, (4) Southeast Asia: Philippines, Sri Lanka, Thailand, Indonesia, Vietnam, Cambodia, Laos, Malaysia, Maldives, Burma, (5) High-income Asia pacific: Brunei, Singapore, (6) North Africa and Middle East: Jordan, Kuwait, Lebanon, Oman, Afghanistan, Yemen, Bahrain, Iran, Iraq, United Arab Emirates, Qatar, Saudi Arabia, Syria, Egypt, Palestine, Turkey, (7) Central Europe: North Macedonia, Poland, Romania, Croatia, Czechia, Bosnia and Herzegovina, Montenegro, Albania, Bulgaria, Hungary, Slovakia, Slovenia, Serbia, (8) Eastern Europe: Republic of Moldova, Russia, Ukraine, Estonia, Lithuania, Belarus, Latvia, and (9) Western Europe: Cyprus, Greece, Israel. To ensure replicability and transparency of results, our study follows the Guidelines for Accurate and Transparent Health Estimates Reporting (GATHER) (Supplementary Appendix A) (17).

### Statistical analyzes

The absolute numbers and ASR of mortality and DALYs for stroke attributed to dietary risks were calculated in “B&R” countries. For estimated metrics, the 95% uncertainty interval (UI) were reported, and 95% UI was calculated by drawing 1,000 times from each number of posterior distributions, using the 2.5th and 97.5th ordering of the uncertainty distribution (1). ASR, as a weighted mean of the age-specific rates, were estimated using a global age structure from 2019, which allow comparisons across time, countries and subregions. We focused on three specific age groups: 20 to 54 years, 50 to 74 years and ≥ 75 years and 6 dietary risk factors (high in sodium, high in red meat, low in fruits, low in vegetables, low in fiber and low in whole grains). The temporal trends of disease burden were assessed using average annual percent change (AAPC) by using the Joinpoint regression software from 1990 to 2019, 95% confidence intervals (CIs) for trend segment identified. The annual ASR of diet-induced stroke mortality and DALYs was designated as a dependent variable, and the year was assigned as an independent variable. The Heteroscedastic Errors Option was set to constant variance, the maximum number of joinpoints was set at 5, and the Log-linear model (ln y = xb) was chosen. For segmented line regression, the Bayesian information criterion (BIC) was used to calculate the optimal number of change points. The software uses a *Z*-test to check whether the slope for each trend segment is significantly different from prior segment (18).

In addition, we evaluated AAPCs of age-standardized DALYs for stroke, stratified by sex, age and specific dietary factors. Meanwhile, we compared the changes in AAPC of stroke burden attributed to dietary risk factors in the last decade and throughout the study period (1990–2019 and 2010–2019). If both the AAPC estimate and the lower limit of 95% UI were positive, ASR of mortality and DALYs showed an upward trend. Conversely, if both the AAPC estimate and the upper limit of the 95% UI were negative, then ASR of mortality and DALYs exhibited a decreasing trend (setting 3% as the cut-off point and ≥ 3% as a larger decrease). Other than that, ASR was considered to be stable (19). All analysis was conducted using the Joinpoint Regression Program (Version 4.9.0.0, The National Cancer Institute, MD, United States) (20). The map visualization of the “B&R” member states was performed using “ggmap” package in R software (version 4.3.0, R core team). The “ggmap” package is an extension package, which obtains shapefiles from Google Maps.^2^ *p* < 0.05 was considered statistically significant.

## Results

Absolute number of mortality and DALYs in 1990 and 2019 caused by stroke attributed to modifiable dietary risk factors in the “B&R” member countries are shown in Table 1. In 2019, the number of stroke deaths and DALYs attributable to dietary risks were 671,872 cases (95% UI 436,354–937,093) and 1.67 million cases (95% UI 1.15–2.24) in China. We found geographical differences in mortality and DALYs of diet-attributable stroke among member countries, with Bulgaria, Hungary, and Serbia being the three highest countries in 1990, Bulgaria, North Macedonia, and Montenegro in Central Asia in 2019. The country with the lowest number of mortality and DALYs is the Qatar in North Africa and Middle East (27 cases, 95% UI, 16–42 and 1716 cases, 95% UI, 1112–2,454) in 2019. From 1990 to 2019, the countries with the largest decreases in the number of diet-induced stroke deaths and DALYs were Czechia and Hungary, and the countries with the largest increases were Albania and Mongolia.

**Table 1:** The absolute number of mortality and DALYs for stroke attributed to dietary risk factors in the “B&R” countries in 1990 and 2019.

Countries	1990				2019			
	Mortality		DALYs		Mortality		DALYs	
	Number	*95%UI*	Number	*95%UI*	Number	*95%UI*	Number	*95%UI*
East Asia								
China	519,444	365,947–684,456	13,921,162	10,116,646–17,796,087	671,872	436,354–937,093	16,729,078	11,517,379–22,374,535
Central Asia								
Armenia	718	483–990	17,050	11,815–23,033	569	372–832	12,110	8,298–17,357
Azerbaijan	1,399	891–1982	36,209	23,396–50,005	2,135	1,289–3,294	52,449	32,653–78,181
Georgia	3,072	1934–4,377	69,738	44,304–97,106	2,364	1,543–3,446	45,212	29,968–63,855
Kazakhstan	6,667	5,090–8,529	168,353	132,157–207,529	6,656	4,814–9,142	163,128	119,957–219,323
Kyrgyzstan	1,666	1,260–2,149	42,090	32,626–53,248	1,252	889–1731	35,033	25,428–47,156
Mongolia	855	641–1,240	25,107	19,183–34,406	1873	1,333–2,538	57,938	41,807–78,194
Tajikistan	961	606–1,343	23,739	15,739–32,543	1,429	885–2,131	38,296	24,443–56,100
Turkmenistan	785	578–1,019	22,466	16,826–28,601	1,635	1,136–2,265	49,060	34,938–66,409
Uzbekistan	4,277	3,058–5,646	113,905	83,509–147,688	6,583	4,406–9,083	200,534	136,918–273,220
South Asia								
Bangladesh	23,369	17,059–31,023	658,112	474,339–870,935	45,776	30,361–64,458	1,130,114	753,143–1,568,966
Bhutan	55	34–84	1,633	1,024–2,431	78	47–120	1950	1,207–2,894
India	108,806	76,011–149,533	3,193,700	2,266,760–4,261,449	174,510	116,473–245,234	4,878,839	3,303,619–6,726,592
Nepal	2,366	1,542–3,526	68,682	45,629–99,984	3,488	2073–5,294	87,713	53,381–129,061
Pakistan	17,619	11,681–24,627	470,608	321,297–640,398	31,622	21,833–44,086	941,775	664,136–1,308,567
Southeast Asia								
Cambodia	2,825	2058–3,747	82,950	61,408–108,405	5,109	3,401–7,015	135,856	93,442–182,872
Indonesia	54,221	37,097–71,978	1,667,014	1,163,148–2,145,745.	97,556	59,670–138,786	2,721,311	1,691,264–3,808,140
Laos	1,416	938–1969	42,389	29,285–57,404	1967	1,191–2,878	57,057	35,395–82,502
Malaysia	4,630	3,141–6,009	134,520	94,922–171,401	5,660	3,327–8,505	160,675	97,942–236,362
Maldives	35	23–48	1,136	773–1,497	41	25–61	1,222	785–1707
Burma	22,675	15,693–31,437	664,973	467,158–918,224	25,754	16,316–35,697	669,267	436,430–931,955
Philippines	5,899	3,957–7,996	182,098	128,081–239,695	23,588	14,885–33,385	727,040	472,675–1,001,384
Sri Lanka	2,834	1813–3,947	73,671	48,915–99,207	3,446	1932–5,666	86,419	51,348–136,071
Thailand	11,222	7,482–15,119	333,518	231,185–435,191	14,312	8,247–22,373	409,927	253,360–617,746
Vietnam	24,948	16,329–35,322	627,551	420,264–858,595	43,166	27,438–60,949	1,109,704	719,539–1,539,709
High-income Asia pacific								
Brunei	39	26–53	1,235	856–1,614	45	29–63	1,482	1,008–1993
Singapore	483	318–652	13,965	9,403–18,499	390	253–559	12,035	7,905–16,686
North Africa and Middle East								
Afghanistan	3,325	2,163–4,689	96,768	65,406–135,387	4,950	3,288–6,999	167,425	113,188–232,267
Bahrain	17	12–24	611	433–829	34	22–51	1,398	935–1991
Egypt	3,244	2,104–4,720	109,798	72,186–157,112	5,249	2,941–8,653	183,803	108,860–284,020
Iran	3,913	2,872–5,200	114,356	86,128–148,118	5,817	4,128–7,778	144,829	103,456–192,654
Iraq	1,659	1,135–2,340	46,668	32,065–65,410	4,274	2,805–6,140	125,402	81,694–178,735
Jordan	290	205–387	8,296	5,980–10,845	648	455–869	19,217	13,793–25,443
Kuwait	50	36–68	2037	1,504–2,629	185	124–260	6,730	4,743–9,055
Lebanon	116	79–168	3,255	2,157–4,601	247	145–356	6,711	4,382–9,455
Oman	127	83–184	4,165	2,745–5,950	163	113–228	5,979	4,252–8,130
Palestine	277	197–367	6,436	4,618–8,494	393	283–520	9,792	7,142–12,830
Qatar	9	6–14	446	295–638	27	16–42	1716	1,112–2,454
Saudi Arabia	1,028	682–1,499	31,438	20,939–44,957	2,348	1,502–3,373	91,954	60,437–130,780
Syria	1,086	745–1,503	33,414	22,882–45,232	1,672	1,125–2,399	47,512	32,535–66,603
Turkey	2,491	1,690–3,548	69,637	46,884–97,124	5,125	3,403–7,354	117,407	76,754–167,557
United Arab Emirates	102	65–158	4,403	2,932–6,507	533	336–826	26,359	17,108–39,491
Yemen	1,616	1,018–2,377	48,729	31,375–71,113	3,581	2,413–5,184	107,727	73,222–151,603
Central Europe								
Albania	1,071	699–1,454	22,240	15,193–29,147	1,620	914–2,507	27,171	16,031–41,820
Bosnia and Herzegovina	1,501	970–2075	37,006	24,550–49,864	1854	1,013–2,940	34,752	19,262–54,363
Bulgaria	9,895	7,028–12,707	223,459	161,984–283,996	9,204	5,987–12,979	170,587	113,102–238,779
Croatia	3,198	2,159–4,252	70,125	48,450–91,615	2,223	1,363–3,293	39,207	24,901–55,719
Czechia	8,278	5,958–10,889	168,042	124,114–214,304	3,478	2,227–4,962	65,399	44,008–89,638
Hungary	8,708	6,304–11,223	202,197	151,708–251,873	4,160	2,701–5,827	86,770	59,055–118,507
Montenegro	371	235–518	7,577	5,066–10,260	526	299–796	9,357	5,499–14,032
North Macedonia	1,395	913–1963	29,651	19,987–40,241	1848	1,067–2,765	35,011	20,428–51,764
Poland	13,513	9,571–18,021	313,397	231,142–408,272	11,865	7,794–16,992	240,386	165,399–329,341
Romania	16,530	11,197–21,945	369,732	256,820–479,508	15,541	9,644–21,994	284,585	183,930–394,641
Serbia	7,767	5,128–10,468	169,368	115,241–225,429	7,120	4,367–10,752	125,306	78,443–188,322
Slovakia	2,581	1822–3,315	59,980	43,799–75,910	1921	1,232–2,808	41,545	27,580–58,793
Slovenia	976	633–1,424	20,816	14,090–29,112	603	364–910	10,416	6,625–14,989
Eastern Europe								
Belarus	4,037	3,177–5,127	99,149	78,742–123,382	3,678	2,594–5,169	82,302	58,537–114,072
Estonia	693	521–889	15,590	11,893–19,510	199	130–292	4,142	2,828–5,871
Latvia	1,597	1,212–2,102	33,220	25,538–42,669	950	638–1,352	17,136	11,904–23,996
Lithuania	998	726–1,343	24,659	18,635–32,219	957	638–1,390	18,738	12,850–26,167
Republic of Moldova	1,086	723–1,570	27,756	18,524–39,376	998	675–1,436	23,594	16,047–33,420
Russia	86,376	63,584–114,847	1,997,368	1,507,553–2,574,674	73,383	49,738–102,945	1,595,601	1,108,276–2,198,646
Ukraine	25,279	19,213–33,295	555,814	428,147–717,875	18,047	12,353–25,098	417,587	293,387–565,884
Western Europe								
Cyprus	150	106–205	2,977	2,144–4,005	156	108–220	2,912	2078–3,923
Greece	3,716	2,693–5,309	62,659	45,622–88,642	3,818	2,677–5,356	53,746	39,134–72,757
Israel	391	245–639	8,284	5,176–13,062	494	314–757	9,592	6,378–14,118

DALYs, disability-adjusted life-years; UI, uncertainty interval.

**Figure 1:**
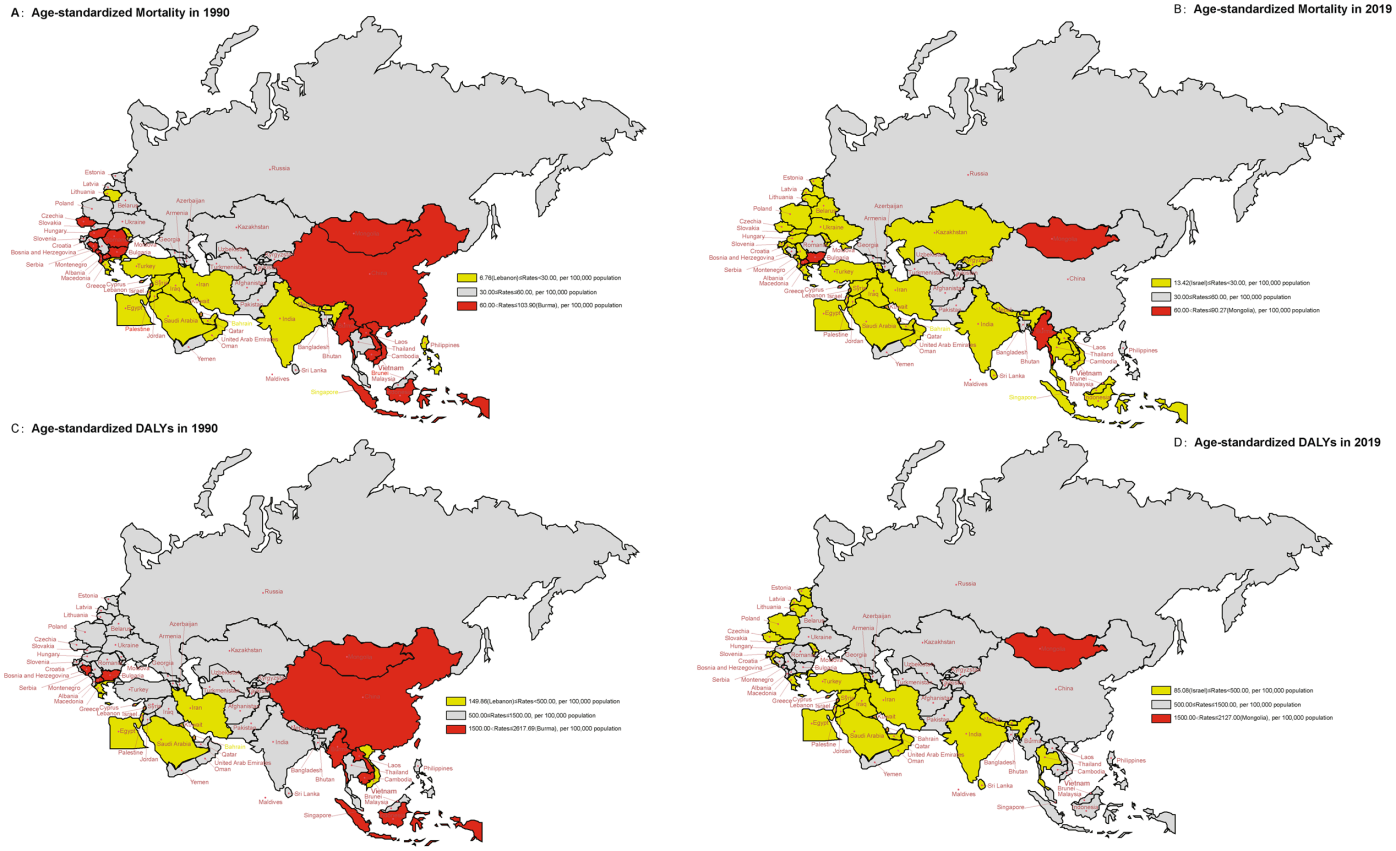
The age-standardized rates of mortality and DALYs due to stroke attributed to dietary risk factors for the “B&R” countries in 1990 and 2019. **(A)** Age-standardized mortality rate in 1990. **(B)** Age-standardized mortality rate in 2019. **(C)** Age-standardized DALYs rate in 1990. **(D)** Age-standardized DALYs rate in 2019.

Figure 1 illustrated the ASR of diet-induced stroke mortality and DALYs in 1990 and 2019 in member countries of the “B&R” initiative. In 1990, the regions with higher age-standardized mortality and DALYs from diet-related stroke were concentrated in Central Europe, East Asia and Southeast Asia. In 1990, the country with the lowest age-standardized mortality and DALYs of diet-induced stroke was Lebanon (6.76 per 100,000 population and 149.86 per 100,000 population, respectively), the highest in Burma (103.90 per 100,000 population and 2617.69 per 100,000 population, respectively). In 2019, Israel enjoyed the lowest age-standardized mortality and DALYs of diet-induced stroke (13.42 per 100,000 population and 85.08 per 100,000 population, respectively), the highest in Mongolia (90.27 per 100,000 population and 2127.00 per 100,000 population, respectively). The age-standardized mortality and DALYs of stroke attributable to dietary risk factors were generally declining in most member states from 1990 to 2019. However, the mortality and DALYs of diet-induced stroke in Mongolia has remained high. See Supplementary Table S1 for more details.

**Figure 2:**
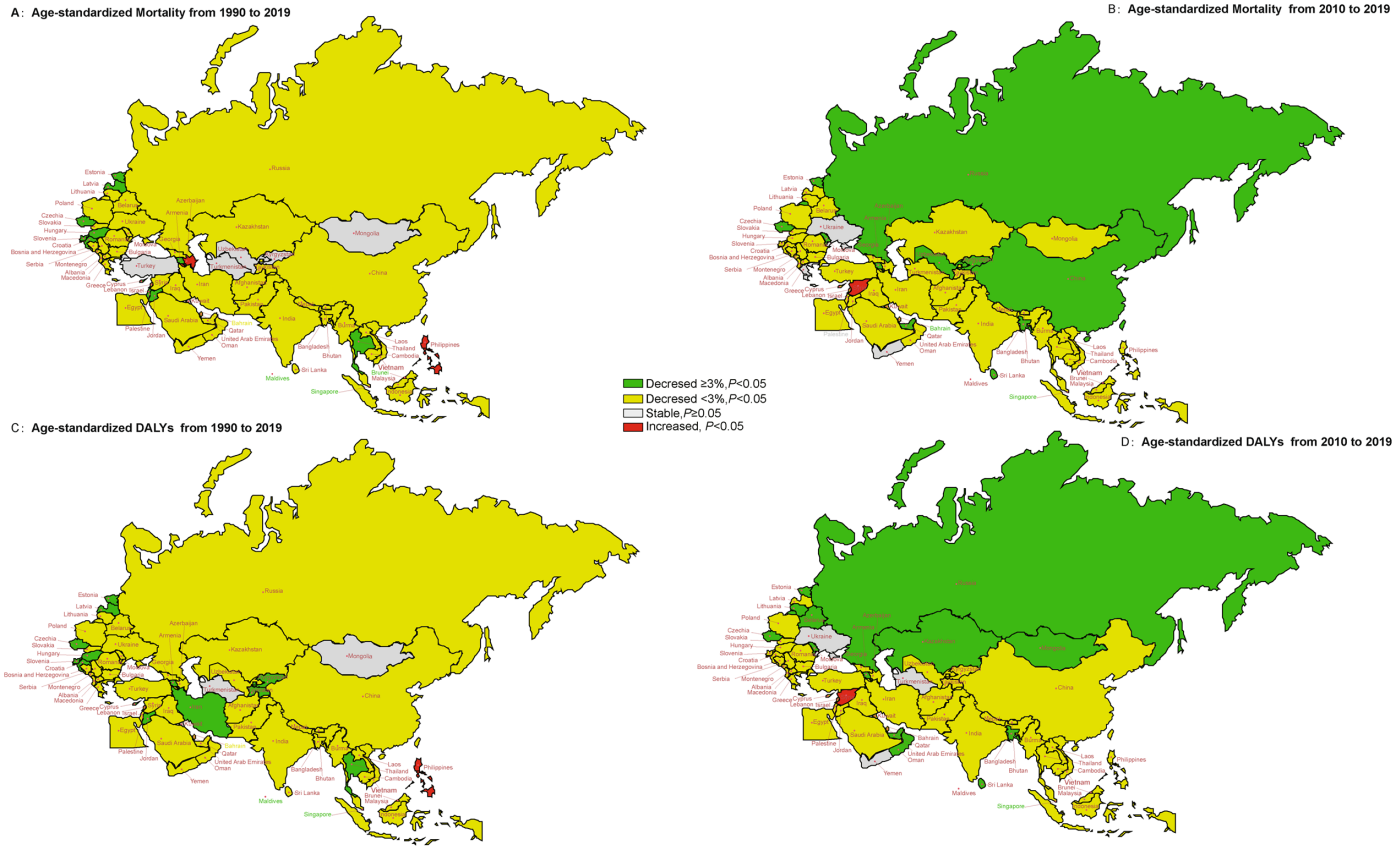
The temporal trend in the age-standardized mortality and DALYs rate of stroke attributed to dietary risk factors for 1990–2019 and 2010–2019 in the “B&R” countries. **(A)** The AAPC of age-standardized mortality from 1990 to 2019. **(B)** The AAPC of age-standardized mortality from 2010 to 2019. **(C)** The AAPC of age-standardized DALYs from 1990 to 2019. **(D)** The AAPC of age-standardized DALYs from 2010 to 2019.

The temporal trend of ASR of mortality and DALYs due to diet-induced stroke for 1990–2019 and 2010–2019 in “B&R” countries was displayed in Figure 2. From 1990 to 2019, the fastest decline in ASR of mortality and DALYs for diet-induced stroke was seen in Estonia, Eastern Europe, with AAPC values of −7.09% (95%CI, −7.72, −6.46%) and − 6.62% (95%CI, −7.20, −6.03%), respectively. However, the AAPC of age-standardized metrics showed an increasing trend in Philippines in Southeast Asia (mortality: AAPC = 1.60%; DALYs: AAPC = 2.03%; *p* < 0.001, respectively). We found no statistically significant differences in AAPC for ASR of diet-induced stroke mortality and DALYs in Kuwait, Mongolia and Turkmenistan in Central Asia and North Africa and Middle East for 1990–2019. In addition, we noted a substantial downward trend in ASR of mortality and DALYs from diet-induced stroke changes in the last decade (2010–2019) for most member states. The ASR of mortality and DALYs due to diet-related stroke in Mongolia remained stable over the full 30 years, yet showed a decreasing trend in the last decade. Trends in age-standardized mortality rates and DALYs for diet-induced stroke in 2010–2019 were not statistically significantly different in Ukraine in Eastern Europe, and Yemen in North Africa and Middle East. See Supplementary Table S2 for more details.

**Figure 3:**
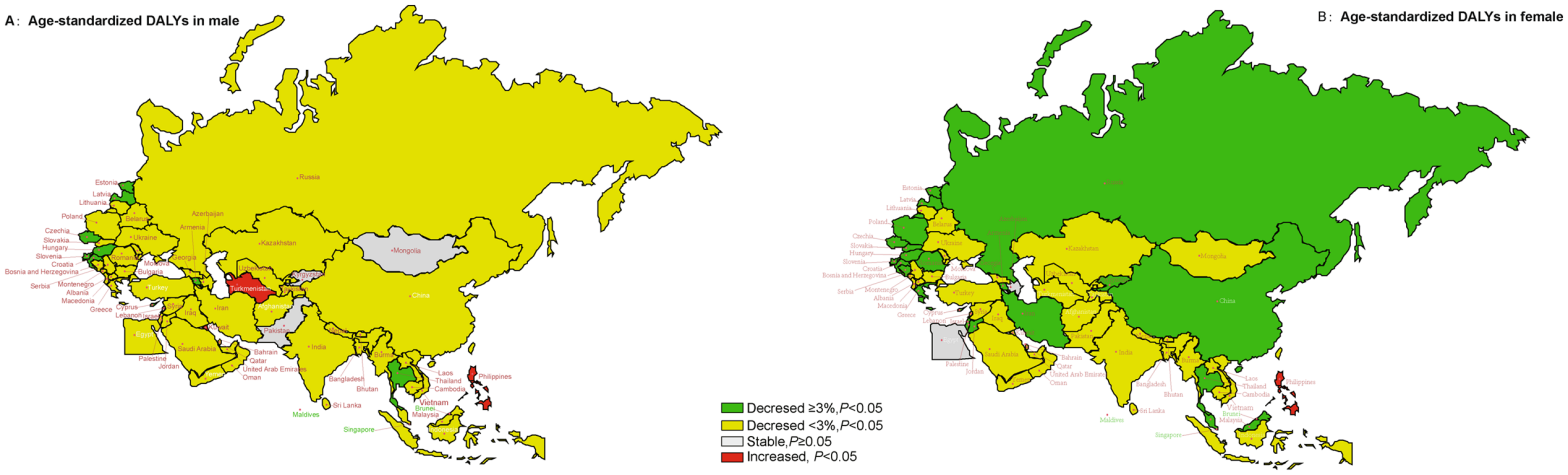
The temporal trend in the age-standardized DALYs rate of stroke attributed to dietary risk factors, stratified by gender for 1990–2019 in the “B&R” countries. **(A)** The AAPC of age-standardized DALYs rate in male. **(B)** The AAPC of age-standardized DALYs rate in female.

Figure 3 illustrated the AAPC values of age-standardized DALYs rate in each member country of the “B&R” in males and females. Most of countries in the “B&R” countries showed a downward trend in AAPC, Estonia had the highest decline in AAPCs of age-standardized DALYs from 1990 to 2019 (male: AAPC = −6.55, 95%CI: −7.15 to −5.95%; female: AAPC = −6.70, 95%CI: −7.27 to −6.13%). However, an upward trend for both sexes in Philippine was observed (mortality: AAPC = 2.31, 95%CI: 1.64–2.99%; DALYs: AAPC = 1.65, 95%CI: 1.00–2.30%; *p* < 0.001, respectively). The ASR of DALYs for diet-induced stroke decreased more in females than in males. For males, the AAPCs in Mongolia, Tajikistan, Pakistan and Lebanon were stable between 1990 and 2019, while the change trend of DALYs was stable for female in Azerbaijan and Egypt (*p* ≥ 0.05) (Supplementary Table S3).

**Figure 4:**
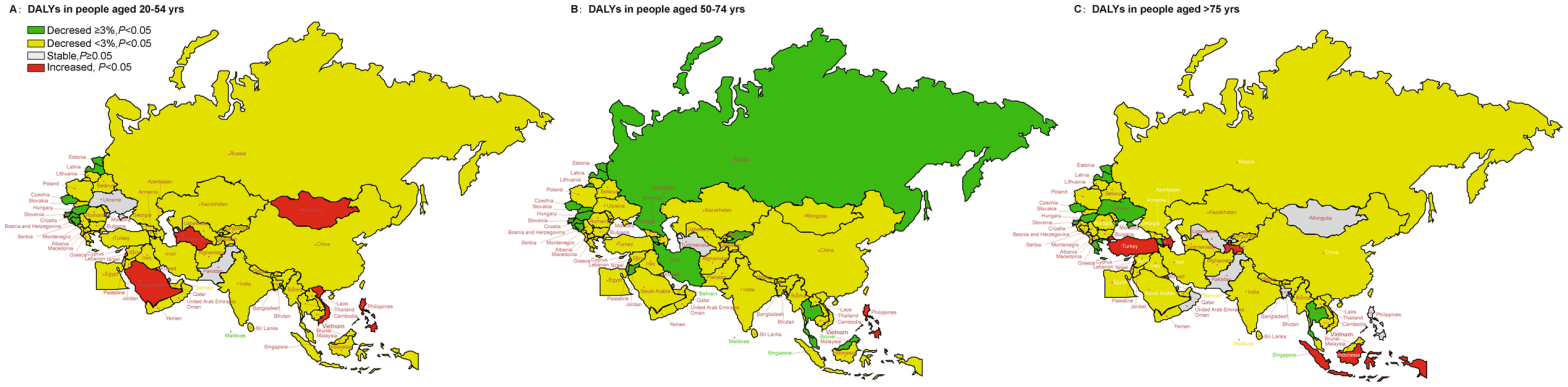
The temporal trend in the DALYs rate of stroke attributed to dietary risk factors, stratified by age for 1990–2019 in the “B&R” countries. **(A)** The AAPC of DALYs rate in people aged 20–54 years. **(B)** The AAPC of DALYs rate in people aged 50–74 years. **(C)** The AAPC of DALYs rate in people aged ≥75 years.

Figure 4 showed the long-term trends of DALYs rate due to diet-related stroke, stratified by age for 1990–2019 for the “B&R” countries. The DALYs rate for stroke attributable to dietary risk factors showed a decreasing trend in all age groups among the member countries in Europe from 1990 to 2019. For people aged 20–54 years, DALYs showed an increasing trend in Mongolia and Turkmenistan in Central Asia, Philippines and Vietnam in Southeast Asia, Saudi Arabia in North Africa and Middle East. Overall, for those aged 50–74, the DALYs for stroke due to dietary risk factors in all other “B&R” member countries showed a decreasing trend, except for the Philippines, which rose (AAPC = 2.13, 95%CI: 1.40–2.87%) and Turkmenistan, which remained stable (AAPC = 0.05, 95%CI: −0.43–0.33%). For adults aged 75 years or older, the AAPC value of DALYs caused by diet-related stroke showed an increasing trend in Azerbaijan, Tajikistan, Indonesia, Kuwait, and Turkey, however, the AAPC values varied steadily in Mongolia, Uzbekistan, Bangladesh, Pakistan, Philippines, Oman and Montenegro, with no statistical significance. See Supplementary Table S4 for more details.

**Figure 5:**
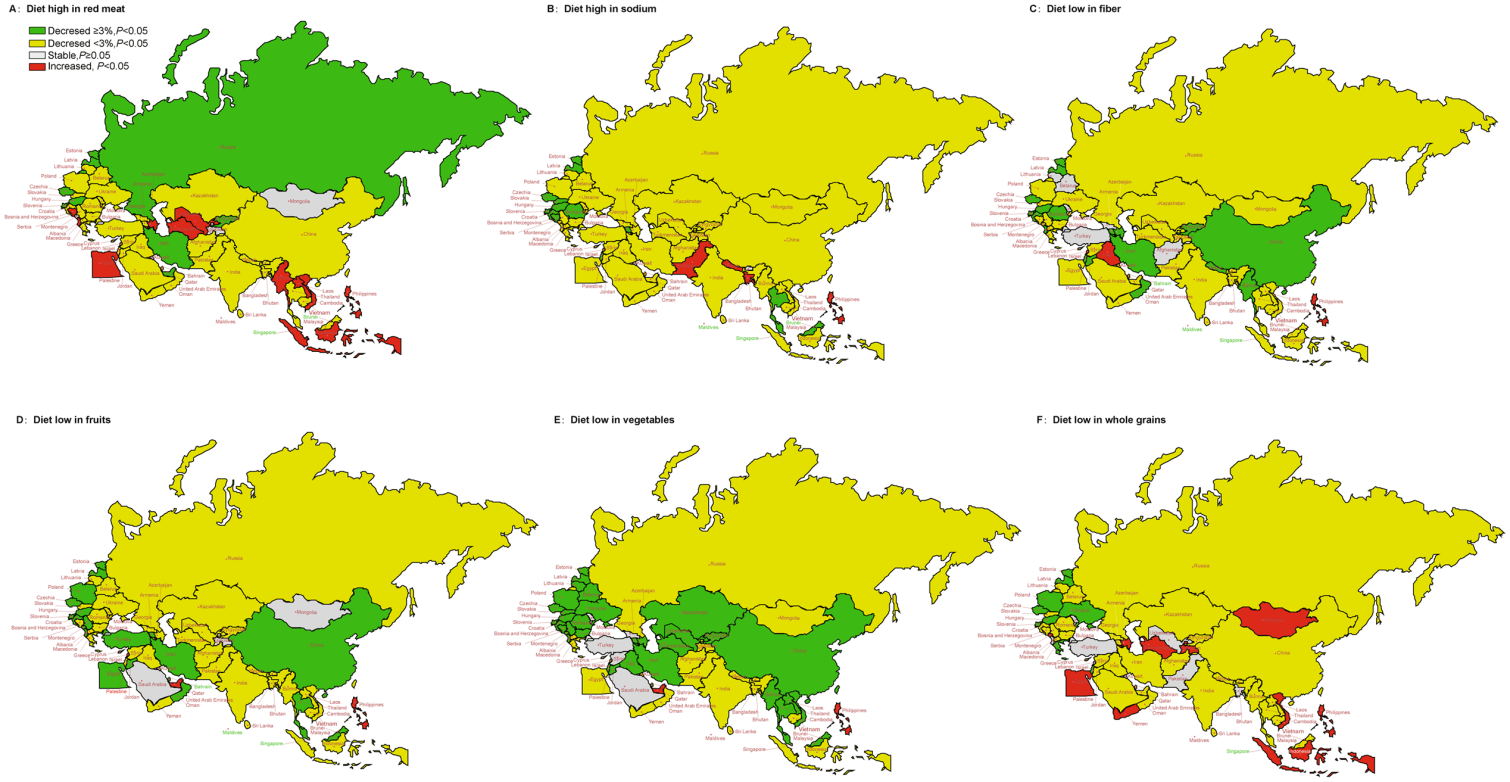
The temporal trend in the DALYs rate of stroke attributed to specific dietary risk factors for 1990–2019 in the “B&R” countries. **(A)** Diet high in red meat. **(B)** Diet high in sodium. **(C)** Diet low in fiber. **(D)** Diet low in fruits. **(E)** Diet low in vegetables. **(F)** Diet low in whole grains.

The AAPC of age-standardized rates for DALYs due to stroke, attributable to specific dietary risk factors for 1990–2019 in the “B&R” member countries was displayed in Figure 5. The ASR of stroke attributable to modifiable dietary risk factors in Philippines significantly increased. Regions with high red meat consumption showed an increasing trend in the ASR of stroke DALYs, such as Azerbaijan, Uzbekistan and Turkmenistan in Central Asia, Indonesia, Laos, Burma, Philippines, Vietnam in Southeast Asia, Egypt in North Africa and Middle East, Albania and Bosnia and Herzegovina in Central Europe. From 1990 to 2019, regions with low fruit and vegetable intake showed a decreasing trend in the ASR of stroke DALYs in “B&R” member countries, except for Philippines and United Arab Emirates. Age-standardized DALYs of stroke showed an increasing trend in regions with low intake of whole grains, such as Mongolia, Azerbaijan, Tajikistan and Turkmenistan in Central Asia, Indonesia, Vietnam and Philippines in Southeast Asia, and Egypt in North Africa and Middle East. See Supplementary Table S5 for more details.

## Discussion

Based on latest data from the GBD Study 2019, we explored the impact of dietary risks on stroke deaths and DALYs in the member states of the “B&R” over past three decade (1990–2019) and the final decade (2010–2019). The results showed geographical differences in mortality and DALYs of diet-attributable stroke among member countries, the age-standardized mortality and DALYs were generally declining in most member states. The decreases in ASRs of diet-related stroke burden may be attributed to decreases in the improvement of living standard, increased awareness of self-health, improved screening programs, the diagnosis of patients in the early stages of disease and better access to effective therapy (21–23). Bulgaria, Hungary, and Serbia were the three countries with the highest diet-attributable ASR of stroke mortality and DALYs in 1990, and Bulgaria, North Macedonia, and Montenegro in Central Asia in 2019. Central Asian countries have seen a shift from traditional Asian to westernized diets, in addition to dietary risks, diabetes, hypertension and cigarette consumption remain the main risk factors for cardiovascular diseases in this region (24). Another possible explanation for this observation was that the highest percentage of total stroke burden was attributable to YLL, suggesting differences in the quality of acute stroke care in these countries. Lack of education and inadequate preventive measures on the treatment of stroke are also key factors contributing to the progressive stroke burden in Central Asia (25). We also found that the mortality and DALYs of diet-induced stroke in Mongolia remained high. It was reported that in Mongolia, low intake of vegetables and fruits resulted in morbidity and mortality of cardiovascular disease that greatly exceed those of Western countries (26). A substantial downward trend in ASR of mortality and DALYs from diet-induced stroke in the final decade (2010–2019) for most member states, compared to the past 3 decades. This phenomenon may be explained by the improvement of stroke awareness in the community, economic development, increased numbers of neurologists, government insurance coverage and so on.

Findings from this study found that the fastest decline in ASR of mortality and DALYs for diet-induced stroke was seen in Estonia, Eastern Europe. Estonia has established detailed stroke registration and management system since the 1970s, and the results of the third population-based stroke register in 2005 showed a decrease in stroke incidence and 28 days case fatality rates compared to the previous decade (27, 28). Furthermore, we noted that the AAPC of age-standardized metrics showed an increasing trend in Philippines in Southeast Asia. A study found that stroke mortality in Philippines has remained high over the past decade, which is similar to our findings (29). In low- and middle-income countries, dietary patterns are changing considerably, such as the replacement of staple-based diets with increased fat, meat and salt intakes (30). Numerous epidemiological studies have focused on dietary habits, as one of the modifiable risk factors and their impact on stroke risk, found a strong association between low-quality diet and stroke risk, however adherence to Mediterranean-style diet pattern has been pointed out to decrease the risk of first stroke (12, 31). Rosato et al. (32) confirmed that dietary patterns of the “B&R” Mediterranean countries exerted a protective effect on the risk of stroke. The Philippines is constantly facing the enormous burden of malnutrition, especially among adults suffering from various forms of malnutrition, and there has also been a noticeable change in food consumption (33, 34). The 2018 Expanded National Nutrition Survey in Philippines analyzed the relationship between food intake and diet quality, and found that the breakfast that Filipinos regularly eat was not nutritious enough (35). This partly explains the increasing trend of diet-induced stroke DALYs in Philippines over the 30 years period.

Meanwhile, our study found that the temporal trends of diet-induced stroke DALYs varied considerably by sex, age and specific dietary risk factors. Females displayed lower negative AAPCs than males in most member counties, suggesting a higher downturn in ASR in females. Gender is a key risk factor for cardiovascular diseases, with biological sex (determined by sex chromosomes and gonadal hormones) and gender (social and cultural behaviors) influencing differences in disease susceptibility and pathology between men and women (36). Thus, this discrepancy is likely due to the different distribution of stroke risk factors between genders and other pathophysiological factors, such as the protective impact of estrogen for females (37). The DALYs rate for diet-induced stroke showed a decreasing trend in all age groups among the member countries in Europe. Dokova et al. (38) found that ASR of stroke DALY declined in West, Central, and East Europe regions and in all twenty East and Central European countries but at a different pace, which is consistent with our study. For those aged 50–74, the DALYs for stroke due to dietary risk factors in all other “B&R” member countries showed a decreasing trend, except for the Philippines, which rose and Turkmenistan, which remained stable. As we have discussed above, inadequate stroke units and rehabilitation facilities, lack of education on stroke prevention and treatment, and traditional diet pattern (high nutrient and fibrous) contribute to the high DALYs of stroke in Central Asia.

Evidence suggested that the main dietary risk factors for deaths and DALYs were low in whole grains, high in sodium, low in fruits and vegetables globally and in many countries (39). Wang et al. (40) demonstrated that ASR of stroke mortality attributable to high sodium intake showed a downward trend from 1990 to 2019 in China, which was consistent with our results. In China, the daily sodium intake was the highest worldwide and started to decrease from 15 g in 1988 (41) to 4.7 g in 2016 (42), therefore, ASR for stroke mortality due to high sodium intake showed a downward trend. This suggests that the implementation of salt reduction policies has had a significant influence on the reduction of stroke mortality and DALYs. It has been observed that age-standardized DALYs of stroke showed an upward tendency in regions with low intake of whole grains, such as Mongolia, Azerbaijan, Tajikistan and Turkmenistan in Central Asia, Indonesia, Vietnam and Philippines in Southeast Asia, and Egypt in North Africa and Middle East, which belonged to low- and middle-income countries. A review article reports that in low- and middle-income countries, consumption of animal-derived foods, oils and sugar is increasing, while consumption of whole grains is low (43). Additionally, dietary fiber is found in fruits and vegetables, and it has been shown to reduce the risk of stroke. A balanced diet is one of the essential elements of a healthy lifestyle, according to studies showing that two-fifths of acute ischemic stroke episodes can be prevented (44). Current comparative risk assessments may significantly underestimate the protective effect of fruit and vegetable intake on stroke. In fact, other factors such as economic income, educational level, and dietary environment have an impact on food choice and diet quality (45). In order to improve diet quality, active collaboration in different areas is necessary given the complexity of dietary practices and the diversity of impacts on diet. Besides, the important contribution of potential interactions or synergistic effects between different dietary risk factors to stroke burden should also be considered. By evaluating and comparing the ASR of diet-induced stroke mortality and DALYs in the “B&R” countries, it is found that different countries face different diet-related stroke challenges. Therefore, under the HSR framework, establishing scientific and effective dietary policy applicable to each country, and improving diet quality will remain the key measures of stroke in “B&R” countries.

Based on the broadest epidemiological dataset available to date, this study analyzes estimates of stroke mortality and DALYs attributable to dietary risk factors from 1990 to 2019, and the corresponding changes in the last decade for the first time. Meanwhile temporal trend in diet-induced stroke DALYs were also explored by sex, age, and specific dietary factors. The main strength is the data collection of “B&R” member countries using the same methods and modeling used in the GBD study. Several limitations exist in this study. Firstly, dietary risks from GBD dataset were not strictly categorized, for example vegetables, fruits and whole grains are all rich in fiber, to some extent they overlap with the fiber group. Also, we did not consider potential interactions or synergistic effects between different dietary risk factors. Secondly, underreporting or misclassification of stroke cases existed in each member country due to different diagnostic criteria, definition and measurement of dietary risk are not the same around the “B&R” countries. Thirdly, given the diversity of whole grain products, it is quite difficult to accurately measure intake, which can lead to measurement errors. Fourthly, while certain confounders (smoking, drinking, BMI) were taken into account in the GBD framework, other variables, such as socioeconomic status and access to healthcare, could still be sources of bias. Finally, our study is based on a secondary analysis of GBD, thus GBD all limitations also apply to our study, which is why age groups were not mutually exclusive in this analysis.

## Conclusion

This study compared stroke mortality and DALYs attributable to dietary risk factors from 1990 to 2019, and the corresponding changes in the last decade, and explored the temporal trend of ASR for diet-induced stroke DALYs stratified by gender, age and specific dietary risk factors in “B&R” countries in the past three decades. We found geographical differences in mortality and DALYs for diet-induced stroke among member countries, with a general downward trend in these indicators from 1990 to 2019 in most member countries. A substantial downward trend in ASR of mortality and DALYs from diet-induced stroke changes in the final decade. Notably, the AAPCs of age-standardized stroke mortality and DALYs attributable to dietary risk factors significantly increased in Philippines. The ASR of DALYs for diet-induced stroke decreased more in females than in males. Therefore, prioritization of public health interventions among “B&R” member countries should be evidence-based and data-driven to address the risks and challenges posed by diet-induced stroke through enhanced health collaboration and resource sharing.

## Data availability statement

The original contributions presented in the study are included in the article/Supplementary material, further inquiries can be directed to the corresponding authors.

## Author contributions

YZ and XL conceived and designed the study. YZ, XL, JW, JZ, and YQ analyzed the data. ZL, JY, WX, HL, CM, XX, and WZ provided advice and consultation. YZ wrote the manuscript. All authors read and approved the submitted manuscript.

## Funding

This work was supported by the Basic Research Program of Shanxi Province (Free exploration) project (20210302123216 to YZ), the Science and Technology Innovation Project of Higher Education Institutions in Shanxi Province (2021L221 to YZ), the Special Disease Construction Project of Pudong Health and Family Planning Commission of Shanghai (Grant No. PWZzb2022-20 to ZL), Discipline Construction Project of Pudong Health and Family Planning Commission of Shanghai (Grant No. PWYts2021-02 to ZL), and the General Program of Health Bureau of the Shanghai (202150015 to ZL). All authors had full access to all data in the study and had responsibility for the decision to submit for publication.

## Conflict of interest

The authors declare that the research was conducted in the absence of any commercial or financial relationships that could be construed as a potential conflict of interest.

## Publisher’s note

All claims expressed in this article are solely those of the authors and do not necessarily represent those of their affiliated organizations, or those of the publisher, the editors and the reviewers. Any product that may be evaluated in this article, or claim that may be made by its manufacturer, is not guaranteed or endorsed by the publisher.

## Footnotes

^1^https://vizhub.healthdata.org/gbd-results/

^2^https://mapsplatform.google.com
