# Supplementary material for: Burden and trends of stroke attributable to dietary risk factors from 1990 to 2019 in the Belt and Road Initiative countries: an analysis from the global burden of disease study 2019
Source: Front Nutr. 2023 Jul 26;10:1235271. doi: 10.3389/fnut.2023.1235271 (PMC10410448; doi:10.3389/fnut.2023.1235271)
Supplement: Supplementary file 1 [file Data_Sheet_1.docx]

| **Table S1** The age-standardized rates of mortality and DALYs due to stroke attributable to dietary risk factors for 1990-2019 in member countries of the "B&R" initiative | | | | | |
| --- | --- | --- | --- | --- | --- |
|  | 1990(per 100,000 people) | |  | 2019 (per 100,000 people) | |
|  | Mortality(95%UI) | DALYs(95%UI) |  | Mortality(95%UI) | DALYs(95%UI) |
| **East Asia** |  |  |  |  |  |
| China | 71.31(49.38, 95.39) | 1604.94(1154.45, 2075.74) |  | 36.31(23.55, 50.85) | 833.77(572.49, 1119.88) |
| **Central Asia** |  |  |  |  |  |
| Armenia | 31.85(21.42, 44.32) | 651.19(448.72, 884.29) |  | 14.32(9.37, 20.96) | 300.76(206.17, 428.73) |
| Azerbaijan | 30.20(19.14, 43.29) | 704.40(452.16, 977.73) |  | 32.80(19.59, 51.24) | 603.19(373.57, 915.38) |
| Georgia | 56.30(35.73, 79.89) | 1162.39(746.66, 1622.44) |  | 36.98(24.36, 53.51) | 787.74(530.32, 1101.26) |
| Kazakhstan | 58.52(44.45, 75.51) | 1315.26(1025.81, 1638.98) |  | 46.03(33.04, 63.39) | 952.18(699.38, 1275.92) |
| Kyrgyzstan | 56.75(42.54, 73.33) | 1365.25(1054.47, 1734.60) |  | 29.47(20.97, 40.84) | 707.27(510.41, 957.59) |
| Mongolia | 86.39(64.76, 124.46) | 2249.22(1706.28, 3125.35) |  | 90.27(64.65, 120.51) | 2127.00(1536.66, 2844.66) |
| Tajikistan | 35.30(22.30, 49.39) | 826.61(544.89, 1142.53) |  | 39.62(24.54, 58.82) | 787.42(490.83, 1153.92) |
| Turkmenistan | 44.32(32.49, 57.93) | 1110.35(828.53, 1419.39) |  | 44.01(30.68, 61.19) | 1142.66(814.63, 1555.97) |
| Uzbekistan | 39.83(28.27, 53.00) | 968.33(705.50,1266.64) |  | 44.36(29.62, 62.90) | 933.24(629.51, 1287.03) |
| **Central Europe** |  |  |  |  |  |
| Albania | 61.94(40.30, 85.27) | 1125.02(760.59, 1494.34) |  | 14.32(9.37, 20.96) | 651.97(389.03, 990.04) |
| Bosnia and Herzegovina | 45.00(28.82, 62.79) | 940.27(615.55, 1280.31) |  | 32.87(17.98, 52.17) | 602.91(339.52, 932.39) |
| Bulgaria | 89.96(63.49, 115.89) | 1854.65(1355.61, 2356.39) |  | 62.18(40.62, 87.82) | 1226.81(822.94, 1715.71) |
| Croatia | 54.60(37.14, 73.16) | 1124.03(773.60, 1474.83) |  | 23.53(14.50, 34.85) | 451.32(291.29, 637.82) |
| Czechia | 62.13(44.80, 81.91) | 1233.47(918.26, 1568.47) |  | 15.77(10.20, 22.45) | 322.89(221.10, 438.49) |
| Hungary | 62.18(45.12, 80.13) | 1417.83(1074.12, 1757.37) |  | 20.67(13.51, 28.87) | 475.88(328.46, 641.83) |
| Montenegro | 86.39(64.76, 124.46) | 1243.17(828.10, 1689.69) |  | 57.36(32.93, 86.92) | 978.78(582.86, 1454.40) |
| North Macedonia | 88.94(57.28, 125.76) | 1671.13(1114.34, 2302.81) |  | 70.67(40.99, 106.35) | 1173.26(692.00, 1735.37) |
| Poland | 33.22(23.34, 44.64) | 733.07(542.78, 954.10) |  | 16.41(10.86, 23.29) | 365.84(254.06, 493.15) |
| Romania | 67.08(45.33, 88.90) | 1361.90(950.49, 1766.88) |  | 39.45(24.80, 55.38) | 784.47(514.86, 1085.47) |
| Serbia | 80.09(52.62, 109.07) | 1587.73(1074.57, 2124.80) |  | 47.83(29.58, 71.61) | 811.41(511.50, 1209.53) |
| Slovakia | 44.83(31.97, 57.51) | 1016.66(744.40, 1284.50) |  | 21.10(13.47, 30.79) | 464.66(311.95, 654.50) |
| Slovenia | 41.28(26.71, 60.37) | 862.44(585.67, 1199.92) |  | 12.06(7.35, 18.07) | 244.00(159.04, 343.36) |
| **Eastern Europe** |  |  |  |  |  |
| Belarus | 32.07(25.23, 40.88) | 774.10(615.24, 960.65) |  | 22.83(16.03, 31.88) | 538.77(385.44, 739.05) |
| Estonia | 34.98(26.31, 44.89) | 776.45(593.97, 968.71) |  | 6.95(4.58, 10.19) | 174.32(122.27, 241.72) |
| Latvia | 45.58(34.60, 59.75) | 939.03(724.42, 1201.70) |  | 21.65(14.71, 30.63) | 452.67(322.64, 626.78) |
| Lithuania | 22.39(16.39, 30.07) | 555.51(421.39, 725.12) |  | 15.32(10.37, 21.94) | 354.79(247.64, 487.95) |
| Republic of Moldova | 27.76(18.80, 40.29) | 631.52(424.66, 894.51) |  | 17.23(11.71, 24.69) | 416.29(284.48, 586.52) |
| Russia | 53.02(38.88, 70.27) | 1128.51(848.89, 1459.85) |  | 31.53(21.42, 44.05) | 708.14(496.22, 966.10) |
| Ukraine | 38.15(28.75, 49.93) | 797.093(611.35,1027.29) |  | 24.29(16.91, 33.43) | 595.16(421.10, 803.74) |
| **High-income Asia pacific** |  |  |  |  |  |
| Brunei | 49.54(32.50, 68.07) | 1137.18(756.07, 1522.75) |  | 20.87(13.41, 29.72) | 473.67(315.49, 653.33) |
| Singapore | 24.84(16.12, 34.03) | 608.05(404.80, 815.12) |  | 5.30(3.42, 7.64) | 152.49(100.35, 211.64) |
| **North Africa and Middle East** | |  |  |  |  |
| Afghanistan | 51.81(34.21, 72.36) | 1336.55(913.46, 1845.00) |  | 41.40(27.48, 59.36) | 1038.60(707.84, 1452.73) |
| Bahrain | 12.85(9.00, 17.58) | 278.62(193.52, 385.86) |  | 5.85(3.87, 8.66) | 123.58(83.57, 180.76) |
| Egypt | 11.93(7.85, 17.48) | 321.20(209.73, 465.02) |  | 8.88(5.02, 14.98) | 250.33(145.11, 390.67) |
| Iran | 19.90(14.57, 26.19) | 431.18(322.35, 560.66) |  | 9.08(6.44, 12.08) | 192.92(136.37, 258.25) |
| Iraq | 22.99(15.78, 32.13) | 555.59(383.08, 784.87) |  | 21.50(14.31, 30.42) | 497.79(325.62, 710.38) |
| Jordan | 28.51(20.26, 37.79) | 614.79(438.96, 805.32) |  | 13.47(9.47, 18.07) | 293.35(209.99, 390.68) |
| Kuwait | 9.34(6.45, 13.08) | 231.58(161.83, 313.83) |  | 7.99(5.14, 11.59) | 195.01(133.56, 276.94) |
| Lebanon | 6.76(4.59, 9.41) | 149.86(100.43, 210.75) |  | 4.88(2.86, 7.02) | 126.67(82.70, 178.68) |
| Oman | 22.43(14.78, 32.24) | 521.66(344.95, 741.15) |  | 13.23(9.15, 18.37) | 275.41(191.61, 378.36) |
| Palestine | 36.84(26.34, 48.68) | 747.97(536.54, 988.40) |  | 22.48(16.11, 29.64) | 430.40(311.83, 565.63) |
| Qatar | 9.12(5.75, 13.46) | 210.83(136.04, 302.46) |  | 5.14(3.10, 7.98) | 107.20(65.91, 161.05) |
| Saudi Arabia | 20.28(13.33, 29.26) | 455.88(305.74, 656.73) |  | 14.54(9.47, 20.60) | 360.33(239.33, 508.81) |
| Syria | 22.31(15.47, 30.92) | 551.79(379.06, 757.45) |  | 16.85(11.68, 23.66) | 388.93(271.68, 538.62) |
| Turkey | 7.98(5.51, 11.20) | 185.39(125.98, 257.39) |  | 6.28(4.22, 8.91) | 134.08(87.32, 191.21) |
| United Arab Emirates | 26.25(17.21, 40.14) | 608.63(402.64, 909.79) |  | 13.55(8.74, 20.10) | 343.26(223.78, 504.76) |
| Yemen | 38.48(24.64, 54.88) | 901.54(578.04, 1303.42) |  | 30.79(20.95, 44.04) | 714.65(496.09, 1006.83) |
| **South Asia** |  |  |  |  |  |
| Bangladesh | 54.08(39.38, 71.94) | 1274.49(934.51, 1688.74) |  | 38.68(25.68, 53.94) | 845.35(563.80, 1178.73) |
| Bhutan | 26.06(15.88, 38.99) | 610.32(380.13, 906.41) |  | 15.45(9.38, 23.71) | 337.55(206.84, 506.79) |
| India | 27.79(19.54, 38.65) | 652.01(460.72, 885.36) |  | 16.27(10.95, 22.92) | 405.95(275.49, 562.09) |
| Nepal | 27.88(18.10, 41.78) | 669.56(442.22, 983.12) |  | 17.58(10.65, 26.77) | 386.00(234.81, 574.94) |
| Pakistan | 33.26(22.08, 47.22) | 788.09(534.61, 1079.68) |  | 32.03(22.62, 44.96) | 767.60(533.42, 1056.57) |
| **Southeast Asia** |  |  |  |  |  |
| Cambodia | 69.56(50.09, 94.15) | 1716.17(1258.51, 2271.29) |  | 49.04(33.04, 67.82) | 1109.72(759.05, 1493.14) |
| Indonesia | 60.78(40.87, 82.25) | 1537.24(1056.47, 2017.39) |  | 52.70(32.12, 75.86) | 1209.81(751.76, 1699.12) |
| Laos | 74.05(48.28, 104.80) | 1898.98(1288.79, 2580.82) |  | 50.21(30.84, 73.13) | 1205.68(749.86, 1758.70) |
| Malaysia | 53.90(36.00, 71.29) | 1361.62(938.69, 1754.33) |  | 23.66(13.91, 35.95) | 581.06(351.05, 852.50) |
| Maldives | 45.78(28.99, 63.91) | 1138.21(753.56, 1531.42) |  | 15.47(9.20, 23.09) | 353.39(220.27, 503.41) |
| Burma | 103.90(71.72, 142.98) | 2617.69(1835.41, 3593.42) |  | 61.42(38.68, 85.86) | 1396.15(909.54, 1935.51) |
| Philippines | 25.18(16.64, 34.78) | 589.88(408.68, 778.75) |  | 32.11(20.32, 45.55) | 851.46(548.01, 1176.43) |
| Sri Lanka | 32.48(20.58, 46.11) | 684.83(446.69, 936.54) |  | 15.61(8.86, 25.68) | 348.50(208.08, 548.42) |
| Thailand | 34.81(22.94, 48.09) | 869.52(593.31, 1151.65) |  | 14.44(8.36, 22.34) | 413.42(256.69, 617.28) |
| Vietnam | 66.40(43.17, 93.57) | 1537.26(1028.09, 2106.80) |  | 50.96(32.30, 72.16) | 1158.52(744.89, 1607.69) |
| **Western Europe** |  |  |  |  |  |
| Cyprus | 24.94(17.77, 34.21) | 409.75(294.56, 547.94) |  | 9.50(6.60, 13.37) | 161.59(115.21, 218.77) |
| Greece | 26.24(19.04, 37.37) | 428.08(313.03, 600.64) |  | 12.53(8.94, 17.33) | 223.29(163.71, 299.01) |
| Israel | 8.71(5.55, 14.08) | 175.05(109.93, 275.27) |  | 3.92(2.51, 5.99) | 85.08(56.54, 125.14) |

DALYs: disability-adjusted life-years; UI, uncertainty interval

| **Table S2** The age-standardized rates of mortality and DALYs due to stroke attributable to dietary risk factors for 1990-2019 and 2010-2019 in member countries  of the "B&R" initiative | | | | | | | | | | | |
| --- | --- | --- | --- | --- | --- | --- | --- | --- | --- | --- | --- |
|  | **1990-2019** | |  | **2010-2019** | |  | **1990-2019** | |  | **2010-2019** | |
|  | **Mortality** | |  | **Mortality** | |  | **DALYs** | |  | **DALYs** | |
|  | AAPC (95%CI, %) | *P* |  | AAPC (95%CI, %) | *P* |  | AAPC (95%CI, %) | *P* |  | AAPC (95%CI, %) | *P* |
| **East Asia** |  |  |  |  |  |  |  |  |  |  |  |
| China | -2.31(-2.49, -2.14) | <0.001 |  | -3.11(-3.45, -2.77) | <0.001 |  | -2.26(-2.39, -2.13) | <0.001 |  | -2.56(-2.83, -2.29) | <0.001 |
| **Central Asia** |  |  |  |  |  |  |  |  |  |  |  |
| Armenia | -3.70(-4.13, -3.26) | <0.001 |  | -4.05(-5.59, -2.48) | <0.001 |  | -3.47(-3.84, -3.1) | <0.001 |  | -3.79(-5.14, -2.41) | <0.001 |
| Azerbaijan | 0.68(0.28, 1.09) | 0.002 |  | -2.04(-2.6, -1.48) | <0.001 |  | -0.45(-0.76, -0.13) | 0.007 |  | -2.37(-2.6, -2.15) | <0.001 |
| Georgia | -1.42(-1.84, -1.01) | <0.001 |  | -2.16(-3.52, -0.78) | 0.007 |  | -1.45(-1.9, -1.01) | <0.001 |  | -2.47(-3.97, -0.95) | 0.006 |
| Kazakhstan | -2.99(-3.55, -2.43) | <0.001 |  | -1.88(-2.21, -1.54) | <0.001 |  | -1.82(-2.28, -1.35) | <0.001 |  | -2.96(-3.25, -2.67) | <0.001 |
| Kyrgyzstan | -2.99(-3.55, -2.43) | <0.001 |  | -4.03(-4.93, -3.13) | <0.001 |  | -3.09(-3.62, -2.55) | <0.001 |  | -4.14(-4.96, -3.3) | <0.001 |
| Mongolia | 0.09(-0.53, 0.71) | 0.779 |  | -2.86(-3.67, -2.04) | <0.001 |  | -0.38(-0.95, 0.20) | 0.195 |  | -3.00(-3.75, -2.25) | <0.001 |
| Tajikistan | 0.19(-0.33, 0.72) | 0.455 |  | -2.72(-3.09, -2.34) | <0.001 |  | -0.43(-0.8, -0.06) | 0.026 |  | -2.51(-2.87, -2.15) | <0.001 |
| Turkmenistan | 0.03(-0.36, 0.43) | 0.859 |  | -0.70(-1.2, -0.19) | 0.014 |  | 0.22(-0.16, 0.61) | 0.243 |  | -0.63(-1.3, 0.03) | 0.059 |
| Uzbekistan | -0.27(-1.1, 0.57) | 0.518 |  | -3.33(-3.72, -2.94) | <0.001 |  | -0.80(-1.42, -0.18) | 0.014 |  | -2.46(-2.65, -2.27) | <0.001 |
| **South Asia** |  |  |  |  |  |  |  |  |  |  |  |
| Bangladesh | -0.74(-1.26, -0.22) | 0.007 |  | -3.31(-4.62, -1.98) | <0.001 |  | -1.03(-1.4, -0.66) | <0.001 |  | -3.06(-3.88, -2.23) | <0.001 |
| Bhutan | -1.86(-1.94, -1.77) | <0.001 |  | -1.47(-1.53, -1.4) | <0.001 |  | -2.13(-2.22, -2.04) | <0.001 |  | -1.61(-1.7, -1.53) | <0.001 |
| India | -2.04(-2.18, -1.91) | <0.001 |  | -1.64(-1.81, -1.47) | <0.001 |  | -1.74(-1.84, -1.63) | <0.001 |  | -1.65(-1.83, -1.47) | <0.001 |
| Nepal | -1.56(-1.88, -1.25) | <0.001 |  | -0.14(-0.22, -0.06) | 0.003 |  | -1.86(-2.2, -1.53) | <0.001 |  | -0.40(-0.47, -0.34) | <0.001 |
| Pakistan | -0.30(-0.53, -0.07) | 0.012 |  | -1.34(-1.45, -1.23) | <0.001 |  | -0.29(-0.54, -0.04) | 0.024 |  | -1.30(-1.44, -1.16) | <0.001 |
| **Southeast Asia** |  |  |  |  |  |  |  |  |  |  |  |
| Cambodia | -1.42(-1.52, -1.32) | <0.001 |  | -0.86(-0.93, -0.79) | <0.001 |  | -1.75(-1.86, -1.63) | <0.001 |  | -1.11(-1.21, -1.01) | <0.001 |
| Indonesia | -0.31(-0.38, -0.24) | <0.001 |  | -0.86(-1.06, -0.65) | <0.001 |  | -0.68(-0.74, -0.61) | <0.001 |  | -1.12(-1.29, -0.96) | <0.001 |
| Laos | -1.61(-1.77, -1.45) | <0.001 |  | -1.40(-1.74, -1.06) | <0.001 |  | -1.82(-1.99, -1.64) | <0.001 |  | -1.69(-2.01, -1.36) | <0.001 |
| Malaysia | -2.80(-3.04, -2.56) | <0.001 |  | -1.15(-1.8, -0.48) | 0.004 |  | -2.84(-3.13, -2.55) | <0.001 |  | -0.81(-1.33, -0.28) | 0.008 |
| Maldives | -4.41(-4.7, -4.11) | <0.001 |  | -1.59(-1.95, -1.22) | <0.001 |  | -4.71(-5.04, -4.38) | <0.001 |  | -1.46(-1.86, -1.05) | <0.001 |
| Burma | -2.10(-2.25, -1.96) | <0.001 |  | -1.34(-1.59, -1.09) | <0.001 |  | -2.47(-2.63, -2.31) | <0.001 |  | -1.80(-2.08, -1.53) | <0.001 |
| Philippines | 1.60(0.95, 2.26) | <0.001 |  | -1.74(-1.95, -1.54) | <0.001 |  | 2.03(1.37, 2.70) | <0.001 |  | -1.72(-1.88, -1.55) | <0.001 |
| Sri Lanka | -2.14(-2.44, -1.83) | <0.001 |  | -3.97(-4.69, -3.25) | <0.001 |  | -2.15(-2.39, -1.91) | <0.001 |  | -3.31(-3.97, -2.65) | <0.001 |
| Thailand | -3.59(-3.79, -3.38) | <0.001 |  | -1.75(-2.52, -0.98) | 0.001 |  | -3.12(-3.34, -2.9) | <0.001 |  | -1.18(-1.71, -0.65) | 0.001 |
| Vietnam | -0.60(-0.77, -0.43) | <0.001 |  | -2.22(-2.31, -2.14) | <0.001 |  | -0.68(-0.83, -0.54) | <0.001 |  | -2.00(-2.04, -1.96) | <0.001 |
| **High-income Asia pacific** | |  |  |  |  |  |  |  |  |  |  |
| Brunei | -3.20(-3.39, -3.01) | <0.001 |  | -1.24(-1.51, -0.97) | <0.001 |  | -3.28(-3.51, -3.04) | <0.001 |  | -0.75(-1.2, -0.29) | 0.005 |
| Singapore | -5.60(-5.74, -5.46) | <0.001 |  | -4.95(-5.79, -4.1) | <0.001 |  | -5.07(-5.24, -4.89) | <0.001 |  | -3.85(-4.48, -3.22) | <0.001 |
| **North Africa and Middle East** | | <0.001 |  |  |  |  |  |  |  |  |  |
| Afghanistan | -1.04(-1.35, -0.73) | <0.001 |  | -1.61(-1.87, -1.35) | <0.001 |  | -1.13(-1.47, -0.79) | <0.001 |  | -1.82(-2.25, -1.39) | <0.001 |
| Bahrain | -2.66(-2.94, -2.39) | <0.001 |  | -3.57(-4.57, -2.55) | <0.001 |  | -2.94(-3.1, -2.78) | <0.001 |  | -2.67(-3.38, -1.95) | <0.001 |
| Egypt | -0.69(-0.86, -0.51) | <0.001 |  | -0.63(-1.11, -0.14) | 0.017 |  | -0.53(-0.72, -0.34) | <0.001 |  | -0.41(-0.75, -0.08) | 0.021 |
| Iran | -2.97(-3.14, -2.8) | <0.001 |  | -1.43(-1.92, -0.95) | <0.001 |  | -3.00(-3.16, -2.85) | <0.001 |  | -1.21(-1.54, -0.88) | <0.001 |
| Iraq | -0.53(-0.72, -0.33) | <0.001 |  | -1.73(-2.13, -1.34) | <0.001 |  | -0.69(-0.91, -0.47) | <0.001 |  | -1.89(-2.15, -1.63) | <0.001 |
| Jordan | -3.26(-3.6, -2.92) | <0.001 |  | -1.59(-2.27, -0.91) | 0.001 |  | -3.21(-3.55, -2.87) | <0.001 |  | -1.37(-1.75, -0.98) | <0.001 |
| Kuwait | 0.38(-0.32, 1.1) | 0.277 |  | -2.11(-3.51, -0.68) | 0.009 |  | 0.06(-0.56, 0.67) | 0.855 |  | -2.26(-3.31, -1.21) | 0.001 |
| Lebanon | -0.83(-0.98, -0.68) | <0.001 |  | -1.35(-1.51, -1.2) | <0.001 |  | -0.27(-0.45, -0.09) | 0.005 |  | -0.41(-0.53, -0.29) | <0.001 |
| Oman | -1.26(-1.55, -0.96) | <0.001 |  | -1.83(-2.98, -0.66) | 0.007 |  | -1.73(-1.96, -1.5) | <0.001 |  | -3.31(-3.92, -2.69) | <0.001 |
| Palestine | -1.77(-1.95, -1.59) | <0.001 |  | -0.4(-1.12, 0.32) | 0.232 |  | -2.01(-2.19, -1.84) | <0.001 |  | -0.67(-1.24, -0.1) | 0.026 |
| Qatar | -2.02(-2.26, -1.78) | <0.001 |  | -1.85(-2.79, -0.9) | 0.002 |  | -2.52(-2.68, -2.36) | <0.001 |  | -2.80(-3.11, -2.48) | <0.001 |
| Saudi Arabia | -1.01(-1.19, -0.83) | <0.001 |  | -2.71(-2.82, -2.61) | <0.001 |  | -0.54(-0.74, -0.33) | <0.001 |  | -2.50(-2.73, -2.28) | <0.001 |
| Syria | -1.77(-2.12, -1.41) | <0.001 |  | 0.82(0.64, 1.00) | <0.001 |  | -1.92(-2.27, -1.57) | <0.001 |  | 0.98^(0.81, 1.14) | <0.001 |
| Turkey | -0.05(-0.54, 0.44) | 0.824 |  | -2.41(-2.59, -2.23) | <0.001 |  | -0.65(-1, -0.31) | 0.001 |  | -1.92(-2.06, -1.79) | <0.001 |
| United Arab Emirates | -2.43(-2.82, -2.03) | <0.001 |  | -4.78(-5.93, -3.6) | <0.001 |  | -2.10(-2.29, -1.91) | <0.001 |  | -3.07(-3.8, -2.34) | <0.001 |
| Yemen | -1.08(-1.19, -0.96) | <0.001 |  | 0.20(-0.26, 0.66) | 0.354 |  | -1.11(-1.23, -0.99) | <0.001 |  | 0.26(-0.24, 0.77) | 0.266 |
| **Central Europe** |  |  |  |  |  |  |  |  |  |  |  |
| Albania | -1.68(-1.86, -1.49) | <0.001 |  | -0.48(-0.64, -0.31) | <0.001 |  | -1.97(-2.16, -1.78) | <0.001 |  | -0.40(-0.61, -0.19) | 0.002 |
| Bosnia and Herzegovina | -1.29(-1.53, -1.04) | <0.001 |  | -1.23(-1.5, -0.96) | <0.001 |  | -1.87(-2.06, -1.68) | <0.001 |  | -1.42(-1.71, -1.14) | <0.001 |
| Bulgaria | -1.76(-1.97, -1.55) | <0.001 |  | -1.06(-1.56, -0.57) | 0.001 |  | -1.97(-2.18, -1.76) | <0.001 |  | -0.96(-1.46, -0.47) | 0.002 |
| Croatia | -3.22(-3.44, -3.01) | <0.001 |  | -2.72(-3.44, -1.99) | <0.001 |  | -3.53(-3.73, -3.33) | <0.001 |  | -2.86(-3.6, -2.11) | <0.001 |
| Czechia | -5.09(-5.33, -4.84) | <0.001 |  | -3.23(-4.21, -2.24) | <0.001 |  | -4.85(-5.04, -4.67) | <0.001 |  | -3.01(-3.76, -2.25) | <0.001 |
| Hungary | -4.32(-4.53, -4.11) | <0.001 |  | -2.60(-3.26, -1.93) | <0.001 |  | -4.30(-4.51, -4.1) | <0.001 |  | -2.49(-3.04, -1.94) | <0.001 |
| Montenegro | -0.46(-0.67, -0.25) | <0.001 |  | -0.91(-1.44, -0.38) | 0.004 |  | -1.00(-1.26, -0.73) | <0.001 |  | -1.21(-1.5, -0.93) | <0.001 |
| North Macedonia | -1.03(-1.27, -0.8) | <0.001 |  | -1.87(-2.16, -1.59) | <0.001 |  | -1.55(-1.74, -1.36) | <0.001 |  | -1.73(-2.07, -1.39) | <0.001 |
| Poland | -2.85(-3.02, -2.68) | <0.001 |  | -2.55(-3.13, -1.97) | <0.001 |  | -2.73(-2.85, -2.6) | <0.001 |  | -2.43(-2.98, -1.86) | <0.001 |
| Romania | -2.54(-2.87, -2.21) | <0.001 |  | -2.37(-3.06, -1.69) | <0.001 |  | -2.67(-3.02, -2.31) | <0.001 |  | -2.33(-3.09, -1.56) | <0.001 |
| Serbia | -2.17(-2.45, -1.89) | <0.001 |  | -2.25(-2.67, -1.83) | <0.001 |  | -2.88(-3.2, -2.56) | <0.001 |  | -2.65(-3.18, -2.12) | <0.001 |
| Slovakia | -2.64(-2.74, -2.53) | <0.001 |  | -2.21(-2.79, -1.63) | <0.001 |  | -2.73(-2.84, -2.63) | <0.001 |  | -2.31(-2.83, -1.78) | <0.001 |
| Slovenia | -4.63(-4.89, -4.38) | <0.001 |  | -2.53(-3.37, -1.68) | <0.001 |  | -4.82(-5.08, -4.56) | <0.001 |  | -2.30(-3.15, -1.45) | <0.001 |
| **Eastern Europe** |  |  |  |  |  |  |  |  |  |  |  |
| Belarus | -1.92(-2.41, -1.42) | <0.001 |  | -2.81(-3.94, -1.66) | 0.001 |  | -2.00(-2.52, -1.48) | <0.001 |  | -3.25(-4.48, -1.99) | <0.001 |
| Estonia | -7.09(-7.72, -6.46) | <0.001 |  | -3.48(-5.3, -1.64) | 0.003 |  | -6.62(-7.2, -6.03) | <0.001 |  | -3.09(-4.35, -1.81) | 0.001 |
| Latvia | -3.33(-3.67, -2.99) | <0.001 |  | -2.11(-2.42, -1.81) | <0.001 |  | -3.36(-3.73, -2.98) | <0.001 |  | -2.47(-2.88, -2.06) | <0.001 |
| Lithuania | -1.49(-1.79, -1.19) | <0.001 |  | -2.87(-3.30, -2.43) | <0.001 |  | -1.70(-2.03, -1.38) | <0.001 |  | -3.07(-3.7, -2.44) | <0.001 |
| Republic of Moldova | -1.93(-2.34, -1.52) | <0.001 |  | -3.40(-4.43, -2.35) | <0.001 |  | -1.77(-2.26, -1.28) | <0.001 |  | -3.90(-4.9, -2.88) | <0.001 |
| Russia | -2.75(-3.4, -2.09) | <0.001 |  | -3.26(-4.05, -2.47) | <0.001 |  | -2.54(-3.21, -1.87) | <0.001 |  | -3.49(-4.24, -2.74) | <0.001 |
| Ukraine | -2.68(-3.07, -2.29) | <0.001 |  | 0.06(-1.28, 1.42) | 0.918 |  | -2.04(-2.44, -1.64) | <0.001 |  | 1.27(-0.22, 2.78) | 0.085 |
| **Western Europe** |  |  |  |  |  |  |  | <0.001 |  |  | <0.001 |
| Cyprus | -3.86(-4.02, -3.69) | <0.001 |  | -2.83(-3.01, -2.64) | <0.001 |  | -3.77(-3.96, -3.59) | <0.001 |  | -2.48(-2.98, -1.98) | <0.001 |
| Greece | -1.42(-1.84, -1.01) | <0.001 |  | -1.07(-2.15, 0.01) | 0.052 |  | -2.84(-3.09, -2.6) | <0.001 |  | -0.96(-1.83, -0.07) | 0.037 |
| Israel | -3.33(-3.53, -3.12) | <0.001 |  | -1.36(-1.74, -0.99) | <0.001 |  | -2.97(-3.14, -2.8) | <0.001 |  | -1.33(-1.62, -1.04) | <0.001 |

AAPC: average annual percent change; CI: confidence interval

| **Table S3** The temporal trend in the age-standardized DALYs rate of stroke attributed to dietary risk factors, stratified by gender for 1990-2019 in the “B&R” countries | | | | | |
| --- | --- | --- | --- | --- | --- |
|  | Male | |  | Female | |
|  | AAPC (95%CI, %) | *P* |  | AAPC (95%CI, %) | *P* |
| **East Asia** |  |  |  |  |  |
| China | -1.75(-1.86, -1.63) | <0.001 |  | -3.03(-3.19, -2.87) | <0.001 |
| **Central Asia** |  |  |  |  |  |
| Armenia | -3.20(-3.57, -2.82) | <0.001 |  | -3.83(-4.20, -3.46) | <0.001 |
| Azerbaijan | -0.62(-0.91, -0.33) | <0.001 |  | -0.31(-0.65, 0.03) | 0.071 |
| Georgia | -1.08(-1.55, -0.60) | <0.001 |  | -2.08(-2.50, -1.66) | <0.001 |
| Kazakhstan | -1.48(-1.96, -1.00) | <0.001 |  | -2.23(-2.67, -1.78) | <0.001 |
| Kyrgyzstan | -2.63(-3.14, -2.12) | <0.001 |  | -3.74(-4.30, -3.18) | <0.001 |
| Mongolia | 0.41(-0.20, 1.01) | 0.18 |  | -1.25(-1.82, -0.68) | <0.001 |
| Tajikistan | -0.20(-0.48, 0.09) | 0.171 |  | -0.73(-1.20, -0.26) | 0.003 |
| Turkmenistan | 0.67(0.23, 1.10) | 0.004 |  | -0.40(-0.74, -0.06) | 0.022 |
| Uzbekistan | -0.62(-1.23, -0.02) | 0.044 |  | -1.05(-1.70, -0.41) | 0.002 |
| **South Asia** |  |  |  |  |  |
| Bangladesh | -0.88(-1.27, -0.49) | <0.001 |  | -1.16(-1.51, -0.81) | <0.001 |
| Bhutan | -1.68(-1.74, -1.62) | <0.001 |  | -2.59(-2.73, -2.45) | <0.001 |
| India | -1.60(-1.73, -1.48) | <0.001 |  | -1.86(-2.02, -1.70) | <0.001 |
| Nepal | -1.23(-1.57, -0.90) | <0.001 |  | -2.58(-2.92, -2.32) | <0.001 |
| Pakistan | -0.24(-0.52, 0.04) | 0.085 |  | -0.37(-0.59, -0.15) | 0.002 |
| **Southeast Asia** |  |  |  |  |  |
| Cambodia | -1.38(-1.46, -1.31) | <0.001 |  | -2.04(-2.18, -1.90) | <0.001 |
| Indonesia | -0.25(-0.34, -0.16) | <0.001 |  | -1.12(-1.21, -1.04) | <0.001 |
| Laos | -1.72(-1.88, -1.57) | <0.001 |  | -1.95(-2.16, -1.75) | <0.001 |
| Malaysia | -2.74(-3.05, -2.42) | <0.001 |  | -3.00(-3.26, -2.73) | <0.001 |
| Maldives | -4.17(-4.48, -3.85) | <0.001 |  | -5.60(-5.94, -5.26) | <0.001 |
| Burma | -2.32(-2.43, -2.20) | <0.001 |  | -2.56(-2.79, -2.33) | <0.001 |
| Philippines | 2.31(1.64, 2.99) | <0.001 |  | 1.65(1.00, 2.30) | <0.001 |
| Sri Lanka | -1.90(-2.20, -1.61) | <0.001 |  | -2.30(-2.48, -2.11) | <0.001 |
| Thailand | -3.02(-3.30, -2.74) | <0.001 |  | -3.34(-3.50, -3.19) | <0.001 |
| Vietnam | -0.42(-0.57, -0.26) | <0.001 |  | -1.26(-1.39, -1.13) | <0.001 |
| **High-income Asia pacific** |  |  |  |  |  |
| Brunei | -3.08(-3.46, -2.70) | <0.001 |  | -3.33(-3.52, -3.14) | <0.001 |
| Singapore | -5.15(-5.31, -4.98) | <0.001 |  | -5.03(-5.22, -4.84) | <0.001 |
| **North Africa and Middle East** |  |  |  |  |  |
| Afghanistan | -1.11(-1.46, -0.76) | <0.001 |  | -1.09(-1.42, -0.76) | <0.001 |
| Bahrain | -2.85(-3.06, -2.64) | <0.001 |  | -2.99(-3.14, -2.84) | <0.001 |
| Egypt | -0.74(-0.92, -0.56) | <0.001 |  | -0.19(-0.40, 0.02) | 0.073 |
| Iran | -2.86(-2.98, -2.73) | <0.001 |  | -3.11(-3.30, -2.91) | <0.001 |
| Iraq | -0.53(-0.71, -0.35) | <0.001 |  | -0.88(-1.15, -0.61) | <0.001 |
| Jordan | -2.68(-2.89. -2.48) | <0.001 |  | -3.63(-4.12, -3.13) | <0.001 |
| Kuwait | 0.90(0.27, 1.53) | 0.007 |  | -1.44(-2.05, -0.84) | <0.001 |
| Lebanon | 0.19(-0.06, 0.44) | 0.132 |  | -0.64(-0.77, -0.51) | <0.001 |
| Oman | -1.85(-2.08, -1.61) | <0.001 |  | -1.54(-1.76, -1.32) | <0.001 |
| Palestine | -2.05(-2.18, -1.92) | <0.001 |  | -1.98(-2.21, -1.76) | <0.001 |
| Qatar | -2.74(-2.93, -2.55) | <0.001 |  | -1.76(-1.97, -1.55) | <0.001 |
| Saudi Arabia | -0.45(-0.67, -0.22) | <0.001 |  | -0.70(-0.89, -0.51) | <0.001 |
| Syria | -1.74(-2.06, -1.42) | <0.001 |  | -2.00(-2.38, -1.62) | <0.001 |
| Turkey | -0.50(-0.86, -0.15) | 0.007 |  | -0.80(-1.16, -0.45) | <0.001 |
| United Arab Emirates | -2.21(-2.41, -2.01) | <0.001 |  | -1.99(-2.48, -1.49) | <0.001 |
| Yemen | -1.24(-1.36, -1.11) | <0.001 |  | -1.00(-1.11, -0.89) | <0.001 |
| **Central Europe** |  |  |  |  |  |
| Albania | -2.05(-2.29, -1.80) | <0.001 |  | -2.01(-2.16, -1.87) | <0.001 |
| Bosnia and Herzegovina | -1.51(-1.72, -1.29) | <0.001 |  | -2.26(-2.44, -2.08) | <0.001 |
| Bulgaria | -1.77(-1.99, -1.56) | <0.001 |  | -2.15(-2.38, -1.92) | <0.001 |
| Croatia | -3.52(-3.72, -3.31) | <0.001 |  | -3.66(-3.88, -3.45) | <0.001 |
| Czechia | -4.96(-5.15, -4.76) | <0.001 |  | -4.85(-5.04, -4.66) | <0.001 |
| Hungary | -4.21(-4.42, -4.01) | <0.001 |  | -4.40(-4.62, -4.17) | <0.001 |
| Montenegro | -1.11(-1.34, -0.89) | <0.001 |  | -0.88(-1.21, -0.55) | <0.001 |
| North Macedonia | -1.39(-1.61, -1.17) | <0.001 |  | -1.74(-1.92, -1.56) | <0.001 |
| Poland | -2.29(-2.44, -2.13) | <0.001 |  | -3.29(-3.42, -3.16) | <0.001 |
| Romania | -2.39(-2.79, -1.99) | <0.001 |  | -3.01(-3.31, -2.70) | <0.001 |
| Serbia | -2.57(-2.89, -2.25) | <0.001 |  | -3.19(-3.53, -2.86) | <0.001 |
| Slovakia | -2.47(-2.61, -2.33) | <0.001 |  | -3.07(-3.19, -2.94) | <0.001 |
| Slovenia | -4.91(-5.17, -4.66) | <0.001 |  | -4.83(-5.13, -4.54) | <0.001 |
| **Eastern Europe** |  |  |  |  |  |
| Belarus | -1.58(-2.15, -1.02) | <0.001 |  | -2.45(-2.91, -2.00) | <0.001 |
| Estonia | -6.55(-7.15, -5.95) | <0.001 |  | -6.70(-7.27, -6.13) | <0.001 |
| Latvia | -3.29(-3.71, -2.87) | <0.001 |  | -3.52(-3.87, -3.16) | <0.001 |
| Lithuania | -1.32(-1.69, -0.96) | <0.001 |  | -2.10 (-2.38, -1.82) | <0.001 |
| Republic of Moldova | -1.32(-1.81, -0.83) | <0.001 |  | -2.33(-2.82, -1.84) | <0.001 |
| Russia | -2.26(-2.97, -1.55) | <0.001 |  | -3.00(-3.60, -2.39) | <0.001 |
| Ukraine | -1.52(-1.95, -1.09) | <0.001 |  | -2.78(-3.13, -2.43) | <0.001 |
| **Western Europe** |  |  |  |  |  |
| Cyprus | -3.42(-3.66, -3.17) | <0.001 |  | -4.19(-4.41, -3.96) | <0.001 |
| Greece | -2.51(-2.73, -2.30) | <0.001 |  | -3.16(-3.44, -2.88) | <0.001 |
| Israel | -2.90(-3.08, -2.72) | <0.001 |  | -3.12(-3.30, -2.94) | <0.001 |

AAPC: average annual percent change; CI: confidence interval

| **Table S4** The average annual percentage change (AAPC) of DALY rates for stroke attributed to dietary risk factors, stratified by age for 1990-2019 in the “B&R” countries | | | | | | | | |
| --- | --- | --- | --- | --- | --- | --- | --- | --- |
|  | **20-54** | |  | **50-74** | |  | **≥75** | |
|  | AAPC (95%CI, %) | *P* |  | AAPC (95%CI, %) | *P* |  | AAPC (95%CI, %) | *P* |
| **East Asia** |  |  |  |  |  |  |  |  |
| China | -1.14(-1.34, -0.95) | <0.001 |  | -2.58(-2.71, -2.46) | <0.001 |  | -1.95(-2.13, -1.77) | <0.001 |
| **Central Asia** |  |  |  |  |  |  |  |  |
| Armenia | -1.75(-2.18, -1.31) | <0.001 |  | -3.84(-4.43, -3.24) | <0.001 |  | -3.69(-4.08, -3.29) | <0.001 |
| Azerbaijan | -1.00(-1.29, -0.71) | <0.001 |  | -1.80(-2.31, -1.27) | <0.001 |  | 1.33(0.86, 1.79) | <0.001 |
| Georgia | -0.82(-1.48, -0.14) | 0.019 |  | -1.79(-2.34, -1.24) | <0.001 |  | -1.01(-1.41, -0.61) | <0.001 |
| Kazakhstan | -1.63(-2.41, -0.85) | <0.001 |  | -2.22(-2.85, -1.59) | <0.001 |  | -1.36(-1.65, -1.08) | <0.001 |
| Kyrgyzstan | -2.68(-3.25, -2.10) | <0.001 |  | -3.32(-3.98, -2.66) | <0.001 |  | -2.94(-3.38, -2.49) | <0.001 |
| Mongolia | 0.77(0.31, 1.24) | 0.002 |  | -0.68(-1.25, -0.10) | 0.024 |  | -0.27(-1.06, 0.52) | 0.488 |
| Tajikistan | -1.52(-1.69, -1.34) | <0.001 |  | -1.48(-2.03, -0.94) | <0.001 |  | 1.28(0.66, 1.91) | <0.001 |
| Turkmenistan | 1.79(1.28, 2.29) | <0.001 |  | -0.05(-0.43, 0.33) | 0.802 |  | -0.46(-0.81, -0.10) | 0.013 |
| Uzbekistan | -0.66(-0.98, -0.33) | <0.001 |  | -1.65(-2.30, -1.01) | <0.001 |  | 0.12(-0.82, 1.06) | 0.799 |
| **South Asia** |  |  |  |  |  |  |  |  |
| Bangladesh | -0.92(-1.05, -0.80) | <0.001 |  | -1.02(-1.34, -0.70) | <0.001 |  | -0.54(-1.17, 0.08) | 0.087 |
| Bhutan | -2.60(-2.79, -2.41) | <0.001 |  | -2.22(-2.30, -2.14) | <0.001 |  | -1.28(-1.36, -1.19) | <0.001 |
| India | -0.78(-0.91, -0.65) | <0.001 |  | -1.59(-1.73, -1.44) | <0.001 |  | -2.70(-3.00, -2.39) | <0.001 |
| Nepal | -2.38(-2.78, -1.99) | <0.001 |  | -1.83(-2.21, -1.45) | <0.001 |  | -1.10(-1.34, -0.86) | <0.001 |
| Pakistan | -0.15(-0.40, 0.10) | 0.235 |  | -0.51(-0.77, -0.25) | <0.001 |  | -0.11(-0.28, 0.07) | 0.21 |
| **Southeast Asia** |  |  |  |  |  |  |  |  |
| Cambodia | -1.84(-2.00, -1.67) | <0.001 |  | -2.07(-2.21, -1.92) | <0.001 |  | -0.80(-0.87, -0.72) | <0.001 |
| Indonesia | -0.59(-0.67, -0.50) | <0.001 |  | -0.89(-0.97, -0.81) | <0.001 |  | 0.20(0.12, 0.27) | <0.001 |
| Laos | -1.71(-1.91, -1.51) | <0.001 |  | -2.24(-2.44, -2.05) | <0.001 |  | -1.22(-1.34, -1.10) | <0.001 |
| Malaysia | -2.00(-2.34, -1.67) | <0.001 |  | -3.22(-3.60, -2.85) | <0.001 |  | -2.20(-2.39, -2.00) | <0.001 |
| Maldives | -5.01(-5.50, -4.52) | <0.001 |  | -5.61(-5.94, -5.27) | <0.001 |  | -2.93(-3.16, -2.70) | <0.001 |
| Burma | -2.32(-2.53, -2.12) | <0.001 |  | -2.87(-3.08, -2.66) | <0.001 |  | -1.28(-1.38, -1.17) | <0.001 |
| Philippines | 3.97(3.10, 4.84) | <0.001 |  | 2.13(1.40, 2.87) | <0.001 |  | 0.41(-0.03, 0.85) | 0.064 |
| Sri Lanka | -1.05(-1.32, -0.77) | <0.001 |  | -2.44(-2.61, -2.26) | <0.001 |  | -1.77(-2.14, -1.40) | <0.001 |
| Thailand | -0.76(-1.04, -0.49) | <0.001 |  | -3.77(-4.05, -3.49) | <0.001 |  | -3.34(-3.50, -3.19) | <0.001 |
| Vietnam | 0.70(0.45, 0.96) | <0.001 |  | -1.27(-1.35, -1.18) | <0.001 |  | -0.42(-0.61, -0.23) | <0.001 |
| **High-income Asia pacific** |  |  |  |  |  |  |  |  |
| Brunei | -2.35(-2.72, -1.98) | <0.001 |  | -3.98(-4.36, -3.59) | <0.001 |  | -2.49(-2.65, -2.34) | <0.001 |
| Singapore | -2.99(-3.22, -2.76) | <0.001 |  | -5.64(-5.91, -5.36) | <0.001 |  | -4.67(-4.80, -4.54) | <0.001 |
| **North Africa and Middle East** |  |  |  |  |  |  |  |  |
| Afghanistan | -0.98(-1.31, -0.65) | <0.001 |  | -1.22(-1.63, -0.80) | <0.001 |  | -0.80(-1.01, -0.59) | <0.001 |
| Bahrain | -1.67(-2.01, -1.32) | 0.008 |  | -4.65(-4.88, -4.41) | <0.001 |  | -2.26(-2.61, -1.90) | <0.001 |
| Egypt | -0.60(-0.87, -0.33) | <0.001 |  | -0.32(-0.50, -0.15) | <0.001 |  | -0.52(-0.71, -0.33) | <0.001 |
| Iran | -2.34(-2.66, -2.01) | <0.001 |  | -3.70(-3.95, -3.45) | <0.001 |  | -2.86(-3.03, -2.69) | <0.001 |
| Iraq | -0.69(-0.95, -0.42) | <0.001 |  | -0.91(-1.14, -0.68) | <0.001 |  | -0.19(-0.33, -0.06) | <0.001 |
| Jordan | -2.47(-2.77, -2.18) | <0.001 |  | -3.60(-4.09, -3.11) | <0.001 |  | -2.64(-2.89, -2.39) | <0.001 |
| Kuwait | 0.08(-0.41, 0.58) | 0.742 |  | -0.33(-1.07, 0.41) | 0.371 |  | 0.62(0.06, 1.18) | 0.031 |
| Lebanon | 0.19(-0.06, 0.44) | 0.137 |  | -0.01(-0.15, 0.13) | 0.861 |  | -0.60(-0.79, -0.40) | <0.001 |
| Oman | -2.76(-3.09, -2.42) | <0.001 |  | -2.83(-3.20, -2.46) | <0.001 |  | -0.27(-0.87, 0.33) | 0.365 |
| Palestine | -2.01(-2.28, -1.75) | <0.001 |  | -2.80(-2.93, -2.67) | <0.001 |  | -1.51(-1.71, -1.30) | <0.001 |
| Qatar | -3.18(-3.49, -2.87) | <0.001 |  | -3.79(-4.04, -3.54) | <0.001 |  | -1.46(-1.76, -1.17) | <0.001 |
| Saudi Arabia | 0.63(0.41, 0.84) | <0.001 |  | -0.66(-0.92, -0.40) | <0.001 |  | -1.09(-1.22, -0.95) | <0.001 |
| Syria | -1.42(-2.02, -0.83) | <0.001 |  | -2.10(-2.52, -1.69) | <0.001 |  | -1.46(-1.82, -1.11) | <0.001 |
| Turkey | -1.29(-1.52, -1.06) | <0.001 |  | -0.88(-1.17, -0.59) | <0.001 |  | 0.56(0.09, 1.04) | 0.021 |
| United Arab Emirates | -0.80(-1.67, 0.08) | 0.073 |  | -2.57(-2.75, -2.38) | <0.001 |  | -2.06(-2.63, -1.48) | <0.001 |
| Yemen | -1.34(-1.57, -1.11) | <0.001 |  | -1.27(-1.41, -1.12) | <0.001 |  | -0.77(-0.85, -0.70) | <0.001 |
| **Central Europe** |  |  |  |  |  |  |  |  |
| Albania | -0.72(-0.97, -0.47) | <0.001 |  | -2.70(-2.98, -2.42) | <0.001 |  | -1.36(-1.57, -1.15) | <0.001 |
| Bosnia and Herzegovina | -2.03(-2.33, -1.74) | <0.001 |  | -2.54(-2.80, -2.29) | <0.001 |  | -0.54(-0.77, -0.30) | <0.001 |
| Bulgaria | -1.92(-2.24, -1.59) | <0.001 |  | -2.26(-2.56, -1.96) | <0.001 |  | -1.20(-1.34, -1.06) | <0.001 |
| Croatia | -3.53(-3.73, -3.33) | <0.001 |  | -4.10(-4.48, -3.71) | <0.001 |  | -2.52(-2.71, -2.33) | <0.001 |
| Czechia | -4.00(-4.28, -3.72) | <0.001 |  | -5.11(-5.45, -4.77) | <0.001 |  | -4.61(-4.82, -4.40) | <0.001 |
| Hungary | -4.68(-4.95, -4.42) | <0.001 |  | -4.29(-4.59, -4.00) | <0.001 |  | -3.71(-3.90, -3.51) | <0.001 |
| Montenegro | -1.95(-2.44, -1.46) | <0.001 |  | -1.36(-1.83, -0.88) | <0.001 |  | 0.00(-0.20, 0.20) | 0.995 |
| North Macedonia | -1.68(-1.91, -1.45) | <0.001 |  | -2.29(-2.61, -1.97) | <0.001 |  | -0.85(-1.01, -0.70) | <0.001 |
| Poland | -2.67(-2.88, -2.45) | <0.001 |  | -2.67(-2.88, -2.46) | <0.001 |  | -2.93(-3.13, -2.73) | <0.001 |
| Romania | -2.13(-2.53, -1.74) | <0.001 |  | -2.92(-3.43, -2.41) | <0.001 |  | -2.06(-2.34, -1.78) | <0.001 |
| Serbia | -3.40(-3.80, -2.99) | <0.001 |  | -3.77(-4.30, -3.23) | <0.001 |  | -1.65(-1.96, -1.35) | <0.001 |
| Slovakia | -2.62(-2.80, -2.44) | <0.001 |  | -3.15(-3.29, -3.01) | <0.001 |  | -2.10(-2.20, -1.99) | <0.001 |
| Slovenia | -4.68(-4.97, -4.39) | <0.001 |  | -5.31(-5.66, -4.97) | <0.001 |  | -3.91(-4.19, -3.62) | <0.001 |
| **Eastern Europe** |  |  |  |  |  |  |  |  |
| Belarus | -1.33(-1.97, -0.68) | <0.001 |  | -2.43(-3.08, -1.77) | <0.001 |  | -1.62(-1.97, -1.27) | <0.001 |
| Estonia | -6.27(-6.98, -5.55) | <0.001 |  | -6.68(-7.30, -6.05) | <0.001 |  | -6.62(-7.19, -6.05) | <0.001 |
| Latvia | -3.04(-3.56, -2.52) | <0.001 |  | -3.41(-3.88, -2.93) | <0.001 |  | -3.16(-3.43, -2.90) | <0.001 |
| Lithuania | -1.25(-1.73, -0.77) | <0.001 |  | -1.94(-2.29, -1.60) | <0.001 |  | -1.19(-1.47, -0.91) | <0.001 |
| Republic of Moldova | -1.56(-2.30, -0.81) | <0.001 |  | -1.74(-2.33, -1.16) | <0.001 |  | -1.89(-2.17, -1.61) | <0.001 |
| Russia | -1.27(-2.12, -0.42) | 0.005 |  | -3.05(-3.92, -2.18) | <0.001 |  | -2.75(-3.30, -2.20) | <0.001 |
| Ukraine | -0.17(-0.68, 0.34) | 0.498 |  | -2.41(-2.91, -1.91) | <0.001 |  | -3.34(-3.64, -3.04) | <0.001 |
| **Western Europe** |  |  |  |  |  |  |  |  |
| Cyprus | -2.80(-3.02, -2.58) | <0.001 |  | -4.18(-4.51, -3.84) | <0.001 |  | -3.46(-3.60, -3.32) | <0.001 |
| Greece | -0.75(-0.87, -0.64) | <0.001 |  | -2.68(-2.95, -2.40) | <0.001 |  | -3.27(-3.64, -2.91) | <0.001 |
| Israel | -1.81(-1.96, -1.65) | <0.001 |  | -3.53(-3.85, -3.21) | <0.001 |  | -2.51(-2.68, -2.35) | <0.001 |

AAPC: average annual percent change; CI: confidence interval

| **Table S5** The average annual percentage change (AAPC) of age-standardized rates for DALYs due to stroke, attributable to dietary risk factors for 1990-2019 in the “B&R” countries | | | | | | | | | | | | | | | | | |
| --- | --- | --- | --- | --- | --- | --- | --- | --- | --- | --- | --- | --- | --- | --- | --- | --- | --- |
|  | **Diet high in red meat** | |  | **Diet high in sodium** | |  | **Diet low in fiber** | |  | **Diet low in fruits** | |  | **Diet low in vegetables** | |  | **Diet low in whole grains** | |
|  | AAPC (95%CI, %) | *P* |  | AAPC (95%CI, %) | *P* |  | AAPC (95%CI, %) | *P* |  | AAPC (95%CI, %) | *P* |  | AAPC (95%CI, %) | *P* |  | AAPC (95%CI, %) | *P* |
| **East Asia** |  |  |  |  |  |  |  |  |  |  |  |  |  |  |  |  |  |
| China | -0.20(-0.34, -0.06) | 0.006 |  | -2.22(-2.35, -2.08) | <0.001 |  | -4.49(-4.76, -4.22) | <0.001 |  | -3.65(-3.90, -3.41) | <0.001 |  | -10.59(-11.10, -10.07) | <0.001 |  | -0.20(-0.32, -0.07) | <0.001 |
| **Central Asia** |  |  |  |  |  |  |  |  |  |  |  |  |  |  |  |  |  |
| Armenia | -0.91(-1.21 -0.61) | <0.001 |  | -4.33(-4.65, -4.01) | <0.001 |  | -5.00(-5.71, -4.29) | <0.001 |  | -4.92(-5.53, -4.30) | <0.001 |  | -8.06(-9.11, -6.99) | <0.001 |  | -2.64(-2.95, -2.34) | <0.001 |
| Azerbaijan | 2.29(1.68, 2.91) | <0.001 |  | -0.85(-1.18, -0.53) | <0.001 |  | -3.63(-4.39, -2.86) | <0.001 |  | -0.89(-1.37, -0.41) | <0.001 |  | -7.41(-9.08, -5.71) | <0.001 |  | 1.58(1.20, 1.97) | <0.001 |
| Georgia | -1.53(-1.89, -1.16) | <0.001 |  | -2.75(-3.05, -2.44) | <0.001 |  | -0.86(-1.84, 0.13) | 0.085 |  | -0.91(-1.56, -0.26) | 0.008 |  | -0.37(-1.59, 0.86) | 0.542 |  | -0.36(-0.67, -0.05) | 0.025 |
| Kazakhstan | -0.90(-1.22, -0.58) | <0.001 |  | -2.57(-2.94, -2.21) | <0.001 |  | -2.75(-3.74, -1.75) | <0.001 |  | -2.46(-3.08, -1.83) | <0.001 |  | -9.00(-10.37, -7.61) | <0.001 |  | -1.12(-1.53, -0.70) | <0.001 |
| Kyrgyzstan | -3.14(-3.58, -2.70) | <0.001 |  | -0.48(-0.55, -0.40) | <0.001 |  | -4.51(-5.27, -3.74) | <0.001 |  | -2.91(-3.54, -2.28) | <0.001 |  | -6.31(-7.49, -5.12) | <0.001 |  | -1.83(-2.25, -1.41) | <0.001 |
| Mongolia | -0.03(-0.58, 0.52) | 0.906 |  | -1.34(-1.80, -0.88) | <0.001 |  | -0.88(-1.53, -0.21) | 0.012 |  | -0.24(-0.83, 0.36) | 0.418 |  | -1.34(-1.99, -0.67) | <0.001 |  | 1.02(0.56, 1.48) | <0.001 |
| Tajikistan | -0.17(-0.51, 0.18) | 0.336 |  | -1.23(-1.46, -1.00) | <0.001 |  | -0.58(-1.10, -0.06) | 0.03 |  | -0.17(-0.63, 0.29) | 0.452 |  | -2.84(-4.55, -1.10) | 0.002 |  | 1.85(1.47, 2.22) | <0.001 |
| Turkmenistan | 2.22(1.71, 2.72) | <0.001 |  | -1.06(-1.43, -0.69) | <0.001 |  | -1.32(-1.83, -0.80) | <0.001 |  | -0.50(-0.92, -0.08) | 0.023 |  | -8.02(-9.53, -6.49) | <0.001 |  | 0.91(0.49, 1.33) | <0.001 |
| Uzbekistan | 0.55(0.23, 0.87) | <0.001 |  | -1.44(-2.00, -0.88) | <0.001 |  | -2.83(-3.87, -1.78) | <0.001 |  | -1.25(-1.94, -0.55) | <0.001 |  | -5.60(-6.95, -4.22) | <0.001 |  | -0.06(-0.68, 0.56) | 0.848 |
| **South Asia** |  |  |  |  |  |  |  |  |  |  |  |  |  |  |  |  |  |
| Bangladesh | -0.23(-0.56, 0.10) | 0.167 |  | 1.11(0.59, 1.64) | <0.001 |  | -1.36(-1.70, -1.02) | <0.001 |  | -1.51(-1.86, -1.15) | <0.001 |  | -1.58(-1.93, -1.22) | <0.001 |  | -0.18(-0.54, 0.18) | 0.316 |
| Bhutan | -1.58(-1.71, -1.44) | <0.001 |  | 0.20(-0.02, 0.43) | 0.076 |  | -3.19(-3.28, -3.10) | <0.001 |  | -2.65(-2.75, -2.54) | <0.001 |  | -2.93(-3.04, -2.82) | <0.001 |  | -0.66(-0.71, -0.61) | <0.001 |
| India | -1.47(-1.62, -1.32) | <0.001 |  | -1.12(-1.23, -1.01) | <0.001 |  | -2.74(-3.00, -2.47) | <0.001 |  | -1.76(-1.89, -1.63) | 0.657 |  | -2.02(-2.12, -1.92) | <0.001 |  | -0.96(-1.07, -0.85) | <0.001 |
| Nepal | -1.17(-1.56, -0.78) | <0.001 |  | 0.69(0.31, 1.07) | <0.001 |  | -4.33(-4.72, -3.94) | <0.001 |  | -2.29(-2.62, -1.96) | <0.001 |  | -2.82(-3.12, -2.52) | <0.001 |  | -0.47(-0.69, -0.24) | <0.001 |
| Pakistan | -0.33(-0.56, -0.09) | 0.008 |  | 1.47(1.21, 1.73) | <0.001 |  | -1.20(-1.32, -1.09) | <0.001 |  | -0.47(-0.76, -0.18) | 0.003 |  | -0.91(-1.15, -0.66) | <0.001 |  | 0.12(-0.15, 0.39) | 0.359 |
| **Southeast Asia** |  |  |  |  |  |  |  |  |  |  |  |  |  |  |  |  |  |
| Cambodia | -1.06(-1.21, -0.92) | <0.001 |  | -2.59(-2.75, -2.44) | <0.001 |  | -1.90(-2.03, -1.77) | <0.001 |  | -1.57(-1.66, -1.48) | <0.001 |  | -1.48(-1.59, -1.37) | <0.001 |  | -0.88(-0.98, -0.77) | <0.001 |
| Indonesia | 0.30(0.14, 0.46) | <0.001 |  | -0.94(-0.99, -0.89) | <0.001 |  | -0.86(-0.99, -0.73) | <0.001 |  | -1.15(-1.20, -1.09) | <0.001 |  | -0.88(-0.96, -0.81) | <0.001 |  | 1.09(0.98, 1.21) | <0.001 |
| Laos | 0.85(0.57, 1.14) | <0.001 |  | -2.28(-2.48, -2.09) | <0.001 |  | -1.84(-2.03, -1.66) | <0.001 |  | -2.61(-2.78, -2.44) | <0.001 |  | -3.01(-3.23, -2.80) | <0.001 |  | -0.58(-0.65, -0.51) | <0.001 |
| Malaysia | -2.82(-3.20, -2.43) | <0.001 |  | -3.20(-3.47, -2.93) | <0.001 |  | -2.79(-3.12, -2.46) | <0.001 |  | -3.26(-3.61, -2.90) | <0.001 |  | -3.32(-3.59, -3.04) | <0.001 |  | -0.83(-1.01, -0.66) | <0.001 |
| Maldives | -1.42(-1.57, -1.28) | <0.001 |  | -5.32(-5.69, -4.95) | <0.001 |  | -4.22(-4.49, -3.96) | <0.001 |  | -4.72(-5.07, -4.36) | <0.001 |  | -6.00(-6.40, -5.61) | <0.001 |  | -2.77(-3.00, -2.53) | <0.001 |
| Burma | 2.51(2.23, 2.79) | <0.001 |  | -2.96(-3.14, -2.78) | <0.001 |  | -3.14(-3.37, -2.91) | <0.001 |  | -2.63(-2.79, -2.46) | <0.001 |  | -3.72(-3.97, -3.47) | <0.001 |  | -0.86(-0.91, -0.80) | <0.001 |
| Philippines | 4.09(3.24, 4.94) | <0.001 |  | 1.05(0.42, 1.69) | 0.002 |  | 0.14(0.08, 0.20) | <0.001 |  | 2.38(1.60, 3.18) | <0.001 |  | 1.75(1.17, 2.33) | <0.001 |  | 1.24(0.84, 1.65) | <0.001 |
| Sri Lanka | -1.35(-1.47, -1.23) | <0.001 |  | -2.70(-2.91, -2.50) | <0.001 |  | -2.88(-3.33, -2.43) | 0.001 |  | -1.52(-1.80, -1.25) | <0.001 |  | -2.38(-2.62, -2.14) | <0.001 |  | -1.23(-1.46, -1.00) | <0.001 |
| Thailand | -1.47(-1.67, -1.26) | <0.001 |  | -3.95(-4.20, -3.70) | <0.001 |  | -2.96(-3.17, -2.74) | <0.001 |  | -3.67(-3.90, -3.43) | <0.001 |  | -3.01(-3.22, -2.80) | <0.001 |  | -1.78(-1.95, -1.61) | <0.001 |
| Vietnam | 2.77(2.37, 3.17) | <0.001 |  | -1.18(-1.33, -1.03) | <0.001 |  | -0.91(-1.07, -0.75) | <0.001 |  | -1.07(-1.23, -0.90) | <0.001 |  | -4.66(-5.26, -4.06) | <0.001 |  | 0.58(0.43, 0.74) | <0.001 |
| **High-income Asia pacific** | |  |  |  |  |  |  |  |  |  |  |  |  |  |  |  |  |
| Brunei | -4.57(-4.99, -4.15) | <0.001 |  | -3.80(-4.04, -3.55) | <0.001 |  | -1.64(-1.79, -1.50) | <0.001 |  | -2.88(-3.10, -2.65) | <0.001 |  | -3.20(-3.46, -2.94) | <0.001 |  | -2.49(-2.65, -2.32) | <0.001 |
| Singapore | -3.63(-3.79, -3.48) | <0.001 |  | -5.63(-5.85, -5.40) | <0.001 |  | -5.25(-5.38, -5.13) | <0.001 |  | -5.77(-5.93, -5.60) | <0.001 |  | -6.73(-6.89, -6.58) | <0.001 |  | -4.83(-4.96, -4.69) | <0.001 |
| **North Africa and Middle East** | |  |  |  |  |  |  |  |  |  |  |  |  |  |  |  |  |
| Afghanistan | -2.49(-2.60, -2.37) | <0.001 |  | -1.15(-1.42, -0.88) | <0.001 |  | -0.11(-0.89, 0.67) | 0.771 |  | -1.64(-2.07, -1.21) | <0.001 |  | -1.53(-1.85, -1.22) | 0.002 |  | 1.12(0.82, 1.43) | <0.001 |
| Bahrain | -2.89(-3.04, -2.74) | <0.001 |  | -2.81(-3.00, -2.61) | <0.001 |  | -3.61(-3.75, -3.47) | <0.001 |  | -3.41(-3.54, -3.29) | <0.001 |  | -4.29(-4.49, -4.10) | <0.001 |  | -2.35(-2.56, -2.14) | <0.001 |
| Egypt | 0.65(0.49, 0.82) | <0.001 |  | -0.14(-0.26, -0.02) | 0.024 |  | -2.46(-3.02, -1.91) | <0.001 |  | -3.13(-3.33, -2.92) | <0.001 |  | -2.11(-2.69, -1.54) | <0.001 |  | 1.54(1.28, 1.79) | <0.001 |
| Iran | -3.12(-3.28, -2.95) | <0.001 |  | -2.36(-2.53, -2.19) | <0.001 |  | -4.33(-4.57, -4.08) | <0.001 |  | -3.52(-3.99, -3.05) | <0.001 |  | -7.48(-8.02, -6.94) | <0.001 |  | -2.16(-2.34, -1.98) | <0.001 |
| Iraq | -1.80(-2.27, -1.33) | <0.001 |  | -0.87(-0.97, -0.78) | <0.001 |  | 1.30(0.40, 2.22) | 0.006 |  | -0.54(-0.82, -0.25) | <0.001 |  | -3.21(-3.90, -2.51) | <0.001 |  | -0.82(-0.89, -0.76) | <0.001 |
| Jordan | -2.88(-3.09, -2.67) | <0.001 |  | -2.95(-3.26, -2.64) | <0.001 |  | -4.30(-4.75, -3.86) | <0.001 |  | -3.09(-3.46, -2.71) | <0.001 |  | -4.06(-4.55, -3.57) | <0.001 |  | -2.73(-3.03, -2.42) | <0.001 |
| Kuwait | -0.05(-0.76, 0.66) | 0.88 |  | 0.55(-0.11, 1.22) | 0.101 |  | -1.16(-1.58, -0.75) | <0.001 |  | 0.51(-0.11, 1.12) | <0.001 |  | -1.75(-2.79, -0.70) | 0.002 |  | 0.11(-0.57, 0.81) | 0.738 |
| Lebanon | -0.62(-0.72, -0.52) | <0.001 |  | -1.17(-1.37, -0.97) | <0.001 |  | 0.86(0.34, 1.39) | 0.002 |  | 2.30(1.94, 2.65) | <0.001 |  | -0.99(-1.24, -0.74) | <0.001 |  | -0.09(-0.27, 0.10) | 0.329 |
| Oman | -0.63(-1.06, -0.20) | 0.005 |  | -1.38(-1.65, -1.11 | <0.001 |  | -4.81(-5.41, -4.21) | <0.001 |  | -3.47(-3.87, -3.07) | <0.001 |  | -2.58(-2.76, -2.39) | <0.001 |  | -0.78(-1.00, -0.56) | <0.001 |
| Palestine | -2.38(-2.55, -2.21) | <0.001 |  | -1.52(-1.61, -1.44) | <0.001 |  | -1.20(-1.32, -1.09) | 0.001 |  | -2.11(-2.24, -1.98) | <0.001 |  | -6.77(-7.61, -5.93) | <0.001 |  | -0.57(-1.12, -0.01) | 0.048 |
| Qatar | -2.15(-2.29, -2.00) | <0.001 |  | -2.21(-2.42, -2.00) | <0.001 |  | -0.92(-1.14, -0.69) | <0.001 |  | -4.21(-4.52, -3.90) | <0.001 |  | -5.00(-5.46, -4.54) | <0.001 |  | -2.22(-2.42, -2.03) | <0.001 |
| Saudi Arabia | -1.11(-1.25, -0.98) | <0.001 |  | -0.86(-1.02, -0.70) | <0.001 |  | -1.10(-1.41, -0.79) | <0.001 |  | 0.10(-0.25, 0.45) | 0.562 |  | -0.15(-0.56, 0.26) | 0.461 |  | -0.44(-0.59, -0.28) | <0.001 |
| Syria | -2.71(-3.01, -2.41) | <0.001 |  | -2.17(-2.47, -1.88) | <0.001 |  | -2.72(-3.27, -2.17) | <0.001 |  | -1.93(-2.33, -1.52) | <0.001 |  | -0.33(-0.79, 0.14) | 0.167 |  | -1.16(-1.43, -0.88) | <0.001 |
| Turkey | -0.64(-1.10, -0.17) | 0.01 |  | -0.59(-0.90, -0.28) | <0.001 |  | 0.15(-0.38, 0.69) | 0.569 |  | -3.58(-3.82, -3.35) | <0.001 |  | -0.32(-0.71, 0.06) | 0.097 |  | 0.25(-0.09, 0.60) | 0.143 |
| United Arab Emirates | -4.46(-4.66, -4.26) | <0.001 |  | -2.30(-2.59, -2.02) | <0.001 |  | -0.94(-1.17, -0.71) | <0.001 |  | 1.19(0.70, 1.68) | <0.001 |  | 6.97(5.91, 8.05) | <0.001 |  | -1.38(-1.79, -0.97) | <0.001 |
| Yemen | -0.59(-0.79, -0.40) | <0.001 |  | -1.28(-1.38, -1.18) | <0.001 |  | -1.33(-1.58, -1.07) | <0.001 |  | -1.85(-2.05, -1.65) | <0.001 |  | -1.66(-1.81, -1.51) | <0.001 |  | 1.02(0.88, 1.17) | <0.001 |
| **Central Europe** |  |  |  |  |  |  |  |  |  |  |  |  |  |  |  |  |  |
| Albania | 1.23(1.00, 1.46) | <0.001 |  | -2.15(-2.36, -1.94) | <0.001 |  | -3.90(-4.36, -3.43) | <0.001 |  | -5.34(-5.78, -4.90) | <0.001 |  | -9.58(-10.26, -6.91) | <0.001 |  | -0.23(-0.50, 0.04) | 0.088 |
| Bosnia and Herzegovina | 0.54(0.32, 0.76) | <0.001 |  | -1.95(-2.14, -1.76) | <0.001 |  | -2.35(-2.85, -1.85) | <0.001 |  | -2.95(-3.17, -2.73) | <0.001 |  | -5.21(-5.65, -4.77) | <0.001 |  | -1.30(-1.47, -1.13) | <0.001 |
| Bulgaria | -1.24(-1.38, -1.10) | <0.001 |  | -2.70(-2.91, -2.48) | <0.001 |  | -1.71(-2.25, -1.17) | <0.001 |  | -1.43(-1.87, -0.99) | <0.001 |  | -5.62(-6.24, -5.00) | 0.482 |  | -0.78(-0.91, -0.65) | <0.001 |
| Croatia | -1.59(-1.98, -1.20) | <0.001 |  | -4.15(-4.34, -3.96) | <0.001 |  | -3.41(-3.67, -3.15) | <0.001 |  | -4.26(-4.55, -3.96) | <0.001 |  | -3.16(-3.44, -2.88) | <0.001 |  | -2.80(-2.97, -2.62) | <0.001 |
| Czechia | -4.81(-5.04, -4.58) | <0.001 |  | -5.46(-5.68, -5.24) | <0.001 |  | -4.26(-4.44, -4.09) | <0.001 |  | -4.11(-4.26, -3.96) | <0.001 |  | -4.39(-4.53, -4.25) | <0.001 |  | -4.49(-4.68, -4.29) | <0.001 |
| Hungary | -3.80(-3.98, -3.63) | <0.001 |  | -4.90(-5.14, -4.65) | <0.001 |  | -3.56(-3.84, -3.27) | <0.001 |  | -3.71(-3.91, -3.51) | <0.001 |  | -6.99(-7.49, -6.48) | <0.001 |  | -3.31(-3.47, -3.15) | <0.001 |
| Montenegro | 0.09(-0.10, 0.27) | 0.339 |  | -1.23(-1.42, -1.03) | <0.001 |  | -2.00(-2.82, -1.17) | <0.001 |  | -3.31(-3.97, -2.63) | <0.001 |  | -3.64(-4.79, -2.47) | <0.001 |  | 1.06(0.80, 1.31) | <0.001 |
| North Macedonia |  |  |  | -1.84(-1.99, -1.69) | <0.001 |  | -1.78(-2.17, -1.39) | <0.001 |  | -1.91(-2.18, -1.64) | <0.001 |  | -2.52(-3.30, -1.74) | <0.001 |  | -0.76(-1.00, -0.52) | <0.001 |
| Poland | -2.46(-2.57, -2.36) | <0.001 |  | -2.15(-2.24, -2.06) | <0.001 |  | -2.74(-3.08, -2.39) | <0.001 |  | -3.32(-3.55, -3.10) | <0.001 |  | -5.10(-5.40, -4.79) | <0.001 |  | -3.41(-3.52, -3.30) | <0.001 |
| Romania | -0.70(-1.10, -0.31) | 0.001 |  | -3.32(-3.67, -2.96) | <0.001 |  | -5.12(-5.60, -4.63) | <0.001 |  | -2.87(-3.34, -2.40) | <0.001 |  | -6.76(-7.48, -6.04) | <0.001 |  | -2.52(-2.81, -2.22) | <0.001 |
| Serbia | -2.19(-2.47, -1.90) | <0.001 |  | -3.17(-3.43, -2.90) | <0.001 |  | -2.97(-3.53, -2.41) | <0.001 |  | -4.04(-4.51, -3.56) | <0.001 |  | -3.47(-4.01, -2.93) | <0.001 |  | -1.56(-1.85, -1.27) | <0.001 |
| Slovakia | -2.07(-2.21, -1.93) | <0.001 |  | -3.36(-3.47, -3.25) | <0.001 |  | -1.76(-2.10, -1.41) | <0.001 |  | -2.93(-3.04, -2.82) | <0.001 |  | -3.71(-3.92, -3.49) | <0.001 |  | -1.73(-1.90, -1.55) | <0.001 |
| Slovenia | -4.59(-4.79, -4.38) | <0.001 |  | -5.19(-5.49, -4.89) | <0.001 |  | -4.95(-5.28, -4.62) | <0.001 |  | -4.84(-5.24, -4.44) | <0.001 |  | -5.00(-5.31, -4.68) | <0.001 |  | -4.38(-4.69, -4.06) | <0.001 |
| **Eastern Europe** |  |  |  |  |  |  |  |  |  |  |  |  |  |  |  |  |  |
| Belarus | -1.34(-1.66, -1.01) | <0.001 |  | -1.67(-2.23, -1.12) | <0.001 |  | -0.21(-1.67, 1.27) | 0.77 |  | -2.36(-2.97, -1.74) | <0.001 |  | -7.48(-8.68, -6.27) | <0.001 |  | -2.60(-3.22, -1.98) | <0.001 |
| Estonia | -5.97(-6.45, -5.48) | <0.001 |  | -6.12(-6.68, -5.55) | <0.001 |  | -8.13(-8.92, -7.34) | <0.001 |  | -6.44(-7.02, -5.86) | <0.001 |  | -8.57(-9.35, -7.78) | <0.001 |  | -6.93(-7.52, -6.34) | <0.001 |
| Latvia | -3.28(-3.58, -2.98) | <0.001 |  | -3.00(-3.41, -2.58) | <0.001 |  | -3.46(-4.12, -2.80) | <0.001 |  | -3.18(-3.63, -2.73) | <0.001 |  | -4.02(-4.76, -3.27) | <0.001 |  | -4.02(-4.30, -3.75) | <0.001 |
| Lithuania | -1.41(-1.76, -1.05) | <0.001 |  | -1.51(-1.83, -1.20) | <0.001 |  | 0.53(-0.26, 1.34) | 0.181 |  | -1.71(-2.05 -1.38) | <0.001 |  | -3.68(-4.10, -3.25) | <0.001 |  | -2.51(-2.75, -2.27) | <0.001 |
| Republic of Moldova | -2.60(-2.86, -2.34) | <0.001 |  | -2.06(-2.48, -1.64) | <0.001 |  | -0.51(-1.51, 0.50) | 0.308 |  | -2.09(-2.68, -1.50) | <0.001 |  | -0.52(-1.69, 0.68) | 0.381 |  | -0.20(-0.62, 0.23) | 0.349 |
| Russia | -3.37(-4.06, -2.67) | <0.001 |  | -1.85(-2.53, -1.17) | <0.001 |  | -2.69(-3.71, -1.66) | <0.001 |  | -2.58(-3.27, -1.89) | <0.001 |  | -0.42(-0.61, -0.22) | <0.001 |  | -2.80(-3.38, -2.22) | <0.001 |
| Ukraine | -1.69(-2.15, -1.23) | <0.001 |  | -1.42(-1.80, -1.05) | <0.001 |  | -1.18(-1.90, -0.44) | 0.003 |  | -1.21(-1.66, -0.76) | <0.001 |  | -5.62(-6.63, -4.60) | <0.001 |  | -3.91(-4.37, -3.44) | <0.001 |
| **Western Europe** |  |  |  |  |  |  |  |  |  |  |  |  |  |  |  |  |  |
| Cyprus | -4.33(-4.58, -4.09) | <0.001 |  | -3.64(-3.80, -3.47) | <0.001 |  | -3.41(-3.58, -3.24) | <0.001 |  | -3.13(-3.36, -2.90) | <0.001 |  | -3.62(-3.89, -3.34) | <0.001 |  | -3.54(-3.70, -3.38) | <0.001 |
| Greece | -2.65(-2.91, -2.40) | <0.001 |  | -2.73(-2.93, -2.53) | <0.001 |  | -1.62(-1.86, -1.37) | <0.001 |  | -2.07(-2.49, -1.65) | <0.001 |  | -1.80(-2.15, -1.44) | <0.001 |  | -3.96(-4.28, -3.64) | <0.001 |
| Israel | -2.45(-2.63, -2.28) | <0.001 |  | -3.31(-3.55, -3.06) | <0.001 |  | -3.26(-3.58, -2.94) | <0.001 |  | -1.90(-2.03, -1.76) | <0.001 |  | -4.59(-5.13, -4.05) | <0.001 |  | -3.50(-3.67, -3.33) | <0.001 |

AAPC: average annual percent change; CI: confidence interval

**GATHER checklist of information included in reports of global health estimates**

| **#** | **Checklist item** | **Section/paragraph/** **interpretation** |
| --- | --- | --- |
| **Objectives and funding** | | |
| 1 | Define the indicators, populations, and time periods for which estimates were made. | Methods / “Data Sources” |
| 2 | List the funding sources for the work. | Funding |
| **Data Inputs** | | |
| *For all data inputs from multiple sources that are synthesized as part of the study:* | | |
| 3 | Describe how the data were identified and how the data were accessed. | As mentioned in the Methods / “Data Sources” section, the details have been published previously. |
| 4 | Specify the inclusion and exclusion criteria. Identify all ad-hoc exclusions. | As mentioned in the Methods / “Data Sources” section, the details have been published previously. |
| 5 | Provide information on all included data sources and their main characteristics. For each data source used, report reference information or contact name/institution, population represented, data collection method, year(s) of data collection, sex and age range, diagnostic criteria or measurement method, and sample size, as relevant. | Available via online data source tools (http://ghdx.healthdata.org/gbd-2019/data-input-sources). |
| 6 | Identify and describe any categories of input data that have potentially important biases (e.g., based on characteristics listed in item 5). | As mentioned in the Methods / “Definitions” section, the details have been published previously. |
| *For data inputs that contribute to the analysis but were not synthesized as part of the study:* | | |
| 7 | Describe and give sources for any other data inputs. | Available via online data source tools (http://ghdx.healthdata.org/gbd-2019/data-input-sources). |
| *For all data inputs:* | | |
| 8 | Provide all data inputs in a file format from which data can be efficiently extracted (e.g., a spreadsheet as opposed to a PDF), including all relevant meta-data listed in item 5. For any data inputs that cannot be shared due to ethical or legal reasons, such as third-party ownership, provide a contact name or the name of the institution that retains the right to the data. | Available via online data source tools (http://ghdx.healthdata.org/gbd-2019/data-input-sources) |
| **Data analysis** | | |
| 9 | Provide a conceptual overview of the data analysis method. A diagram may be helpful. | Flow diagrams of the overall methodological processes were available online  (http://ghdx.healthdata.org/gbd-2019/code/nonfatal-12) |
| 10 | Provide a detailed description of all steps of the analysis, including mathematical formulae. This description should cover, as relevant, data cleaning, data pre-processing, data adjustments and weighting of data sources, and mathematical or statistical model(s). | As mentioned in the Methods / “Statistical Analysis” section, |
| 11 | Describe how candidate models were evaluated and how the final model(s) were selected. | As mentioned in the Methods / “Statistical Analysis” section, the details have been published previously. |
| 12 | Provide the results of an evaluation of model performance, if done, as well as the results of any relevant sensitivity analysis. | As mentioned in the Methods / “Statistical Analysis” section, the details have been published previously. |
| 13 | Describe methods for calculating uncertainty of the estimates. State which sources of uncertainty were, and were not, accounted for in the uncertainty analysis. | Methods / “Statistical Analysis” section |
| 14 | State how analytic or statistical source code used to generate estimates can be accessed. | Methods / “Statistical Analysis” section |
| **Results and Discussion** | | |
| 15 | Provide published estimates in a file format from which data can be efficiently extracted. | Results, and online data tools (data visualization tools, and data query tools,  http://ghdx.healthdata.org/gbd-2019) |
| 16 | Report a quantitative measure of the uncertainty of the estimates (e.g. uncertainty intervals). | Results, and online data tools (data visualization tools, and data query tools,  http://ghdx.healthdata.org/gbd-2019) |
| 17 | Interpret results in light of existing evidence. If updating a previous set of estimates, describe the reasons for changes in estimates. | Discussion, paragraphs 1-4 |
| 18 | Discuss limitations of the estimates. Include a discussion of any modelling assumptions or data limitations that affect interpretation of the estimates. | Discussion, paragraph 5 |
